# Laser ablation in liquids for shape-tailored synthesis of nanomaterials: status and challenges

**DOI:** 10.3762/bjnano.16.137

**Published:** 2025-11-10

**Authors:** Natalie Tarasenka

**Affiliations:** 1 Department of Design, Manufacturing and Engineering Management, University of Strathclyde, 75 Montrose st., G1 1XQ, Glasgow, UK

**Keywords:** laser ablation in liquids, laser irradiation, nanofabrication, nanoparticles, shape control

## Abstract

Shape-and size-controlled synthesis of nanomaterials has been a long-term aim and challenge of modern nanotechnology. Despite many synthesis methods are still mainly focused on the production of near-spherical NPs, a number of emerging applications require nanomaterials of nonspherical shape and developed surface, which determine the functional performance of nanostructured devices. Laser ablation in liquids has been demonstrated as a clean, simple, and versatile NP synthesis method. However, the conditions of NP formation and growth are favouring the production of spherical NPs. There are fewer studies of shape control during laser ablation. With that in mind, this perspective article represents a view on the current stage of the development of laser ablation in liquids from the perspective of shape control of the forming nanomaterials. The key parameters influencing the NP shape are highlighted, including the composition of a liquid, laser focusing conditions and introduction of external fields, and the mechanism of their impact on the conditions for anisotropic NP formation and growth. The description of the methods developed for the control over nanomaterial morphology is summarized by the vision of the current challenges and development routes of laser ablation in liquids.

## Perspective

### 1 Introduction: Mechanisms and key parameters influencing the morphology of laser-produced NPs

The precise control and tailoring of NP parameters has long been an aim of the laser ablation synthesis in liquids. Combining both top-down and bottom-up strategies, laser-assisted methods are demonstrating the prospects to become a versatile nanoscale manufacturing strategy based on clean, sustainable, and large-scale approach applicable to a broad range of nanomaterials [1–11]. The current progress in the pulsed laser ablation in liquid (PLAL) field has been achieved by getting new insights into the process of laser ablation, its stages, and mechanisms allowing to significantly boost the productivity and reach gram-scale NP production [1,4]. Another direction of the research is the control of NP size and size distribution. As a result, the crucial experimental parameters were determined providing strategies to reduce the formation of large NPs and to produce monodisperse small nanocrystals [1,5].

However, the goal of controlled nanomaterials synthesis by PLAL is still not completely achieved. Despite recent advances, there is still a gap in understanding the routes for shape control of the forming nanomaterials hindered by the limited information about the mechanisms of laser ablation process in liquids. The demand for anisotropic nanomaterials is growing, associated with the rapid development of such fields as energy generation and storage, sensing, and catalysis. Another application area of nonspherical NPs stems from the shape dependencies of their optical properties. The shift of plasmonic bands to the near-infrared spectral range observed for high-aspect-ratio nanostructures, such as nanowires, rods, and nanofilaments is extremely demanded for the application in biolabeling and telecom-band laser sources. In conventional wet chemical methods, the controlled formation of nonspherical nanomaterials typically requires complex approaches and addition of chemicals that reduce the sustainability of the approach due to the introduction of purification steps. In PLAL, the conditions created during laser ablation synthesis are favouring the formation and growth of near-spherical NPs. To date, much less research is performed regarding shape-controlled formation of anisotropic materials.

For shape control, the understanding of the underlying mechanisms is required to elucidate the major stages and key parameters to influence the shape of the nanomaterials during synthesis. It is especially relevant when external action is applied, such as temperature, electrical, and magnetic fields, as shown below. However, obtaining the information on spatial and temporal evolution of transient laser-induced plasma represents a challenge due to the short plasma lifetime.

#### 1.1 Plasma formation and propagation

**Figure 1:**
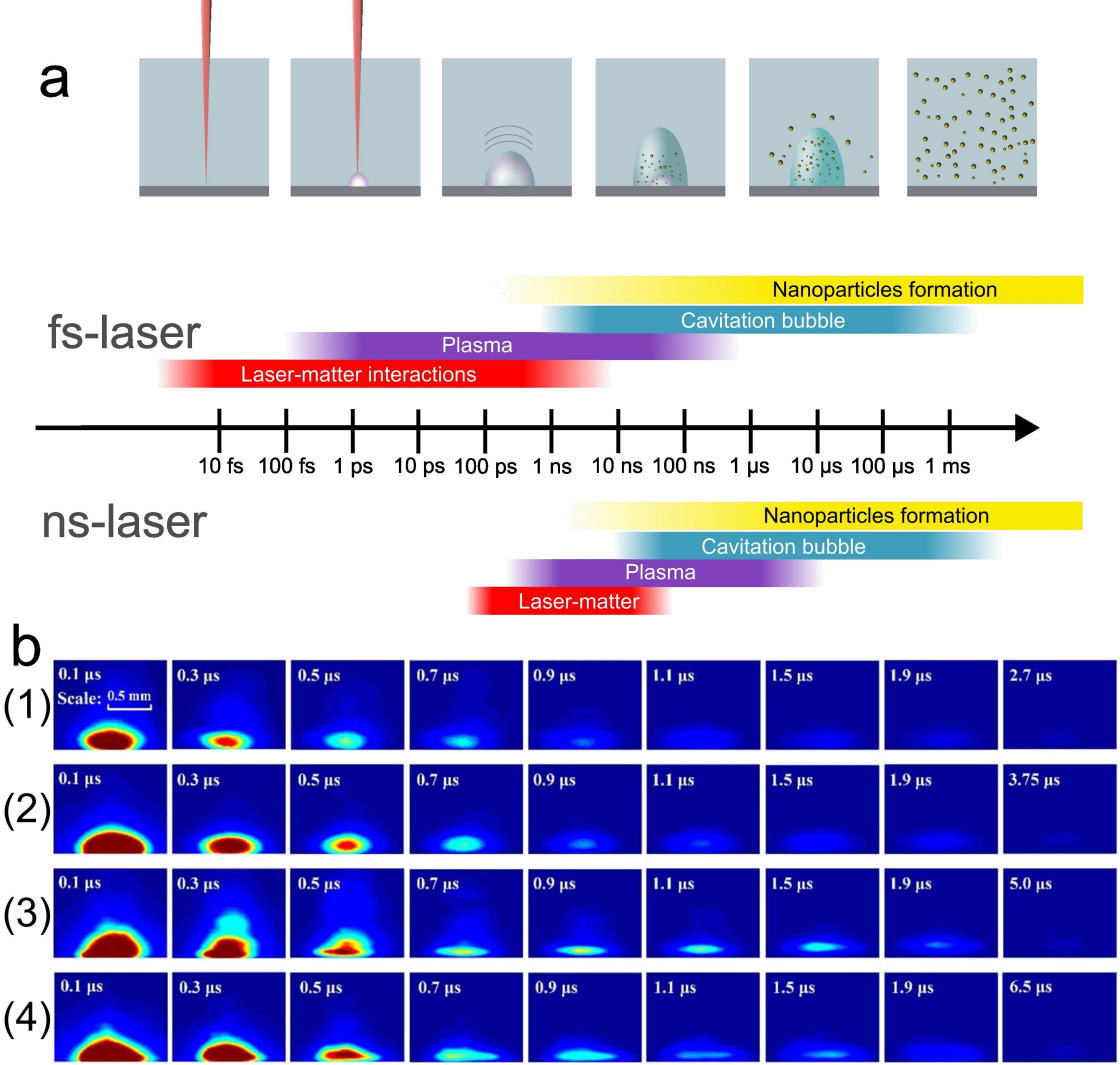
a) Temporal evolution and main stages of laser ablation of a solid target in a liquid for fs and ns pulses. b) Plasma evolution in space-constraint conditions, studied in [6]. (a) Figure 1b was used with permission from [6] (“Spatial restriction on properties of nanosecond pulsed laser ablation of aluminum in water”, by Zhi Zhang et al., *J. Phys. D: Appl. Phys.*, Vol. 53, Article No. 475204, published on 2 September 2020; https://iopscience.iop.org/article/10.1088/1361-6463/abac2c ); © 2020 IOP Publishing Ltd; permission conveyed through Copyright Clearance Center, Inc. All rights reserved. This content is not subject to CC BY 4.0.

In a widely accepted mechanism of PLAL, the formation of NPs occurs via two major processes: thermal evaporation and explosive ejection [1]. According to the thermal evaporation mechanism, the action of a laser beam on the surface of a solid target initiates the absorption of the laser pulse energy. This results in initiation of melting and ionization with the formation of a plasma plume near the target surface. The current understanding of the mechanisms of PLAL distinguishes the process depending on the laser pulse duration (Figure 1a). For the ultrashort pulses (femtosecond duration) the interaction of the ejected material with the laser pulse can be neglected. For longer laser pulses (nanosecond), this interaction should be taken into account, resulting in the ionization and heating of the ejected material.

The initial stages of interaction of a laser beam with a solid target are strongly dependent on laser pulse duration and fluence, surrounding liquid, target morphology and composition, and focusing conditions. The processes occurring during plasma stage are extensively investigated and simulated (Figure 1b) [5–6]. According to the described mechanism, the induction of nonspherical shapes is possible by either variation of the conditions of plasma propagation and expansion or by manipulating the formed seed NPs to induce their anisotropic growth and assembly.

#### 1.2 Cavitation bubble formation, expansion, and collapse

Since plasma plume is confined by a liquid, the subsequent processes involve plasma rapid expansion and cooling by energy transfer to the surrounding liquid, resulting in generation of shockwaves and formation of cavitation bubbles, which typically have a lifetime of several microseconds. During its evolution, the cavitation bubble expands to reach the equilibrium with the confining liquid, after which the shrinking stage begins, which involves the ablated material moving closer to the target surface.

It is typically accepted that release of NPs from the cavitation bubble into a liquid occurs during later stages of cavitation bubble collapse. The latest reports by Dell’Aglio et al. [7], however, demonstrate that particle ejection into a liquid occurs mainly in two steps: in the beginning of the bubble expansion stage, and in the period between bubble collapse and rebound [7]. The major part of the ejected material is released during the second stage of cavitation bubble collapse. The charging of NPs has an impact at this stage, changing the electrostatic pressure, guiding the NP transfer into the solution, and providing the electrostatic repulsion between NPs. Some fraction of NPs can avoid escaping into the solution, remaining trapped in the cavitation bubble and undergoing compression as a result of high pressure in the bubble during collapse. This results in structural reorganization with the formation of aggregated structures comprised by spherical NPs [8].

However, new information on the process continue to appear giving a clearer picture of the process. The latest reports of Spellauge et al. [9] show that the NPs are ejected from the target much earlier than predicted in earlier works. The mechanism suggested by Spellauge et al. [9] involves an explosive phase decomposition of the surface of the target followed by expansion of the plasma plume which is rapidly decelerated by the confining liquid. The formation of NPs according to the explosive ejection mechanism occurs from nano- or microsized droplets or solid fragments directly ejected from the target. The dominating mechanism of NP formation depends on the laser parameters, mainly laser pulse duration and laser fluence. For the nanosecond laser pulse of high energy density (in the range 10^8^–10^10^ W·cm^−2^), the thermal evaporation is preferential [1], while for picosecond and femtosecond laser pulses of lower power density, the explosive ejection mechanism typically prevails resulting in NP formation at earlier stages from the ejected droplets or fragments.

#### 1.3 Nanoparticle nucleation and growth

After the ejection into the liquid, NPs continue their growth. In the case of laser ablation synthesis, the NPs formed in plasma and ejected into the liquid have a spherical morphology, which is explained by a minimal surface energy reached in a spherical shape in the process of plasma quenching. The surface free energy is determined as work per unit area required for the formation of a new surface [12]. Therefore, it is a fundamental parameter that determines nanoparticle growth and stability of different particle shapes. The surface energy is dependent on a multitude of parameters, including NP size, shape, crystal structure, and temperature [12]. As a result, thermodynamically controlled growth typically results in near-spherical NPs. The lowest surface energy of nanospheres also induces the transformation of other shapes into spherical ones. The effect of surface energy is most pronounced in small nanoparticles, typically those with diameters of only a few nanometres, because their high proportion of surface atoms with low coordination and broken bonds leads to increased surface energy [13]. Furthermore, in assembled nanocrystals, the surface energy can be different in different directions, which induces the anisotropic growth and self-assembly of nanostructures with the formation of nanorods and nanosheets. It is currently agreed that media surrounding the particles also influence the surface energy dependence on nanoparticle size. If ions or surfactants are present in the solution, their absorption will further alter the surface energy influencing the preferential growth direction [12].

The spherical shape in laser-produced NPs is also resulting from the thermodynamic equilibrium between the processes of growth and evaporation reached in the plasma bulk [8]. The processes involved in the formation of seeds and NP growth represent thermodynamic condensation and electrostatic growth [8]. As demonstrated in [10], the initially formed seeds are prone to interact with the electrons in plasma, attaining negative charge, which attracts positive ions from plasma enabling growth. This occurs unless the equilibrium is reached with the process of thermodynamic evaporation. As shown in [8], the equilibrium between thermodynamic evaporation and electrostatic growth also determines the typically found spherical morphology of the produced NPs.

**Figure 2:**
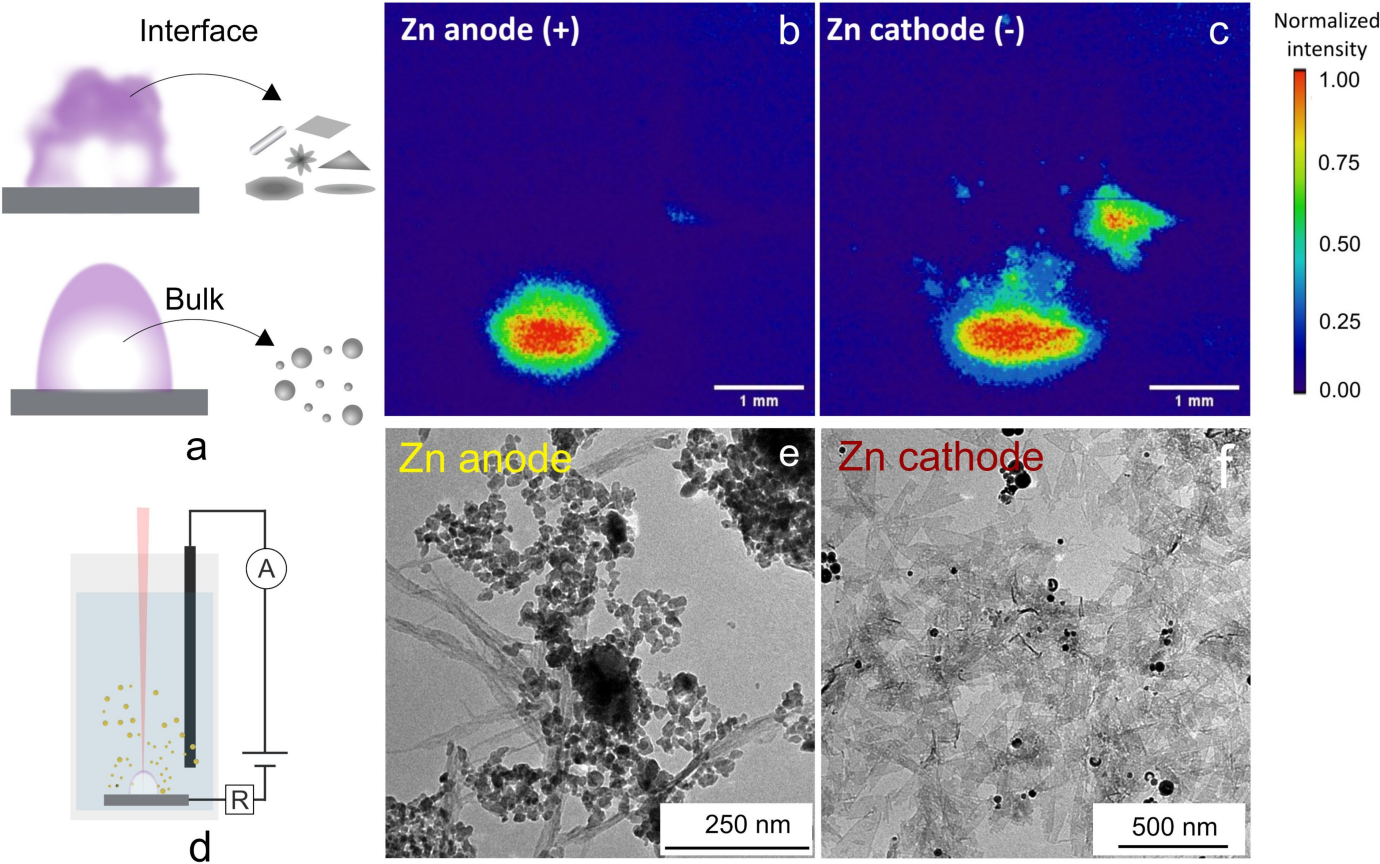
Correlation of plasma plume shape with NP morphology: a) scheme demonstrating the difference in shape of the NPs formed in the bulk and interface of plasma, b,c) imaging of the plasma generated as a result of Zn target ablation in distilled water in the applied electric field with a Zn target connected as an anode (b) and cathode (c); d) scheme of the experimental setup for PLAL in an applied electric field, e,f) TEM images of the NPs, nanowires (e), and nanoflakes (f) formed via PLAL of a Zn anode and cathode, respectively.

The conditions for growth are different for the particles formed at the interface of the plasma and surrounding liquid. Here, the plasma is subjected to fast cooling due to the energy exchange with the surrounding liquid, resulting in nonequilibrium conditions of particle nucleation and growth. This results in the formation of various nanostructure morphologies [8]. Therefore, it can be assumed that manipulating the shape of the laser-induced plasma to enlarge the plasma–liquid interface area may increase the fraction of nonspherical nanoparticles. These conditions can be achieved by application of external electric fields as shown below. As an example, Figure 2 demonstrates the correlation of the shape of laser-induced plasma generated in the electric field applied directly to a Zn target with NP structure. The plasma imaging (Figure 2b,c) demonstrates clear differences in shape depending on the applied field direction. In case of cathode ablation, the plasma is nonuniform as compared to the anode case. As a result, during cathode ablation, spherical nanostructures appear only as a minor component, while ZnO nanosheets dominate in the colloid.

The surface charges can be considered among the key parameters influencing NP shape. The charge of the NPs is influencing the mechanisms of NP formation and growth at the subsequent stages of laser ablation, including the generation of shockwaves, cavitation bubble dynamics, and NP evolution after the release into the colloidal solution. The excessive charge at their surface ensures repulsion and stability of the NP colloid, preventing aggregation [7]. Charge manipulation can be achieved by applying electric fields or varying laser beam polarization. It can also be used at later stages of the NP formation, ensuring self-assembly and growth into oriented structures. In general, the growth of NPs produced by laser ablation in a liquid can follow six mechanisms, as described by Zhang et al. in [14]: LaMer-like growth [15], coalescence [16], Ostwald ripening [17], particle (oriented) attachment [18–21], adsorbate-induced growth [18,22], and reaction-induced growth [23]. Even without applied external fields, the growth processes of the particles via laser ablation are different from the those of conventional wet chemical methods, where particle growth is terminated by reaching some critical seed concentration. The continuous supply of material by every pulse allows to sustain the growth of the NPs throughout the whole ablation process. However, laser ablation is typically performed in the batch liquid reactor. Therefore, interaction of the as-grown particles with the next laser pulses also occurs, which might result in the change of NP size and shape in the photoinduced processes [24] as demonstrated in the next sections.

**Figure 3:**
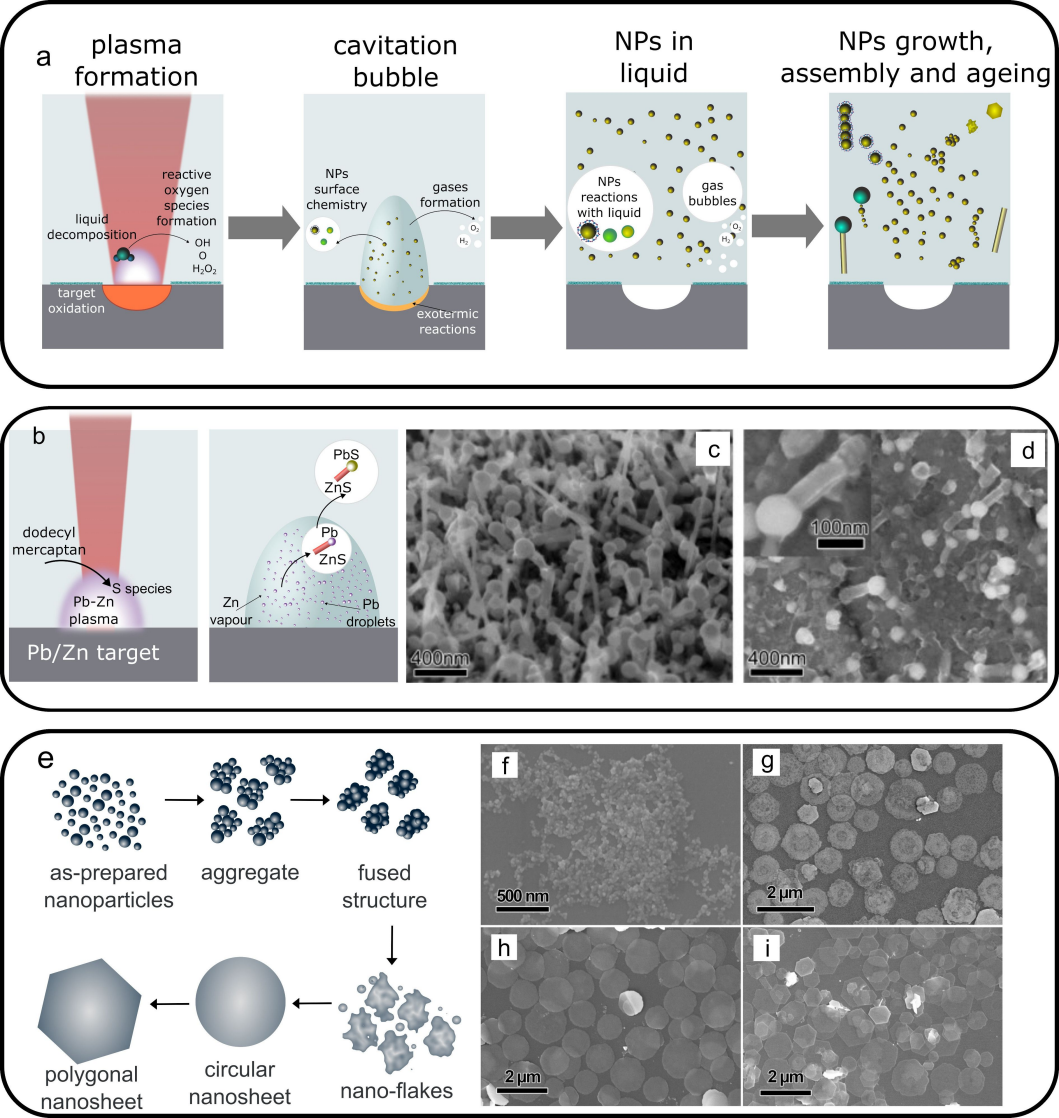
Chemical effects during laser ablation in liquids: a) scheme representing main chemical processes occurring at different stages of laser ablation and nanoparticles formation; b) scheme of PbS/ZnS heterostructures formation by laser ablation in liquid, described in [25], including the stages of generation and expansion of the Zn/Pb metal vapour, formation of Pb nanodroplets, and growth of ZnS nanorods on PbS nanoparticles, c,d) SEM images of the PbS/ZnS heterostructures observed on the surface of Zn/Pb binary alloy target after laser ablation (c) and as-purified PbS/ZnS sample on the aluminum holder (d). Figure 3c,d was reprinted from [25], *Journal of Alloys and Compounds*, Vol. 574, by F. Tian; J. An; H. Cao; S. Guo, “Large scale synthesis of PbS tipped ZnS nanorods heterostructures by long-pulse-width laser ablation in liquid“, Pages 161–164, Copyright (2013), with permission from Elsevier. This content is not subject to CC BY 4.0. (e)–(i) Shape transformation upon ageing: e) scheme demonstrating the mechanism of shape transformation suggested in [26], (f)–(i) FESEM images of PbO nanostructures at different stages of ageing: (f) after 5 min of ageing, (g) 10 min, (h) 20 min, and (i) 24 h. Figure 3e–i was reprinted with permission from [26], Copyright 2020 American Chemical Society. This content is not subject to CC BY 4.0.

Chemical processes occurring during laser ablation influence not only composition but also the chemical structure of the formed nanostructures and their surface, guiding NP growth and morphology change processes. In principle, chemical reactions are present at every stage of laser ablation involving the species ejected from the target and liquid components (Figure 3a). Spatially, the chemical reactions occur in different reaction zones: inside the plasma, cavitation bubble, liquid, or at their interfaces.

The reactions between the ejected target material and liquid molecules are more probable in the liquid phase as compared to the cavitation bubble due to a much higher concentration of the target material species in the bubble as compared to that of the liquid molecules. Moreover, since NPs are shown to be partially localized in the gas bubble and partially travelling with the cavitation bubble front into the liquid, the variation of the fraction of NPs in the bubble and in the liquid can be used for controlling NP size, structure, and oxidation degree, as shown in [27]. This can be done by changing the size and pressure in the cavitation bubble by varying the liquid composition. If more particles are kept inside the cavitation bubble, which can be achieved, for example, as a result of its larger size, it can be expected that nanostructures with lower degree of oxidation and of different structure can be formed. The larger size of the bubble in [27] was a result of the addition of H_2_O_2_ to ethanol that decomposes with the formation of gaseous products, thus increasing the pressure and size of the bubble in accordance with the Rayleigh–Plesset equation. Furthermore, the processes of nuclei growth and coalescence are also slower in the cavitation bubble as compared to that in the liquid phase, which has been shown in [27] to have an impact on the NP size. On the contrary, if the conditions favour more NPs to be transferred into the liquid (and their nucleation and growth is occurring preferentially outside the bubble) then fast passivation of the NP surfaces occurs, which interrupts the coalescence of NPs but favours the irregular growth into anisotropic or coral-shaped particles [27].

The understanding of the occurring chemical reactions in some cases provides the explanation of the regularities regarding NP structure modification. For example, in [28] the formation of iron oxide nanowires was reported only in case of ablation with a 248 nm laser of iron powder suspended in methanol, while other solvents (including water, ethanol, isopropanol, and glycol) resulted in only spherical NPs. Moreover, ablation using a 532 nm laser produced spherical NPs in all the solvents including methanol. The authors explained the influence of the methanol environment on the nanomaterial shape by attributing it to chemical reactions between iron and methanol molecules, which resulted in the formation of goethite (FeO(OH)) and subsequently led to nanowire growth:


[1] $$2\text{Fe}+12{\text{CH}}_{3}\text{OH}\to 6{\text{C}}_{2}{\text{H}}_{5}\text{OH}+2\text{Fe}{\left(\text{OH}\right)}_{3}+3{\text{H}}_{2},$$

[2] $$\text{Fe}{\left(\text{OH}\right)}_{3}\to \text{FeO}(\text{OH})+{\text{H}}_{2}\text{O.}$$

Since iron oxide has an absorption band at 248 nm, the wavelength change from 248 nm to 532 nm resulted in a significant decrease in absorption and consequent production of near-spherical NPs, similarly to those obtained in other solvents that do not directly participate in chemical interactions with the liquid. However, the additional 248 nm laser irradiation of the spherical NPs in methanol could transform NPs into nanowires. This underlines the importance of controlling chemical reactions during all stages of laser ablation and laser modification processes.

Chemical interactions can become key parameters if complex multielement or composite targets are ablated. In this case, the reactions between the species comprising a target and liquid-produced species can determine the resulting morphology and NP structure. In [29], ZnS nanowires were formed by laser ablation of a Cu/Zn target immersed in dodecyl mercaptan using millisecond laser pulses. The authors propose a mechanism in which zinc sulfide is formed via the reactions of zinc species formed in plasma with sulfur-containing reactive species formed as a result of liquid decomposition. The formation of nanowires was only observed in a narrow window of experimental parameters: laser pulse duration in the range 4–5 ms was required. The authors also demonstrated the role of Cu comprising the target. Despite the absence in the formed ZnS nanowire structure, the ablation of a single-element Zn target produced only spherical ZnS@Zn core–shell nanostructures. Similarly, spherical CuS NPs were produced upon ablation of a Cu target in the same experimental conditions. Therefore, the authors suggest the participation of Cu as a catalyst for the growth of ZnS nanowires. Even more complex reaction pathways were suggested by Tian et al. [25] for the formation of PbS-tipped ZnS nanorod heterostructures (Figure 3b–d). The authors also used a binary target, millisecond laser pulses for ablation, and dodecyl mercaptan as the surrounding liquid [25].

Furthermore, the chemical processes occurring during the interaction of plasma with liquid have been found to produce hydrogen and peroxide radicals, as well as hydroxide and hydrogen ions that are changing the liquid parameters (such as pH) and can participate in the processes occurring with NPs during the later stages of growth, self-organization, and ageing. The produced reactive species might result in NP surface modification. The presence of the active ionic species has been shown to guide the formation of Ni(OH)_2_ nanosheets in [30].

After NP formation, the shape transformation can occur during the stages of growth, self-organization, and ageing of the NPs. In this case, laser ablation, which is performed for relatively short periods of time, produces spherical NPs that act as seeds during the growth of nonspherical nanostructures. Such approach has been demonstrated in [26] for the production of PbO nanosheets with thickness less than 15 nm by laser ablation of a lead target in water. After the initial stage, small near-spherical Pb NPs are produced, which are quickly oxidized and assembled into planar structures due to the existence of a preferential [002] growth direction in the PbO structure. The transformation of the morphology occurs after the laser is switched off and the colloid undergoes ageing at room temperature. At an intermediate stage, the particles are organized in a network structure.

The self-assembly processes can result in one-dimensional nanomaterials as well. Yang et al. [31] fabricated ZnSe nanowires through the self-assembly of ≈ 50 nm seed NPs, which were first generated by femtosecond laser ablation of ZnSe powder in water. The transformation to nanowires, which were 100–300 nm in diameter and 50 μm long, was observed during ageing for a week. Furthermore, the authors applied air bubbling through the solution to induce change of nanowires into hollow nanotubes, which is believed to be caused by initiation of hydrolysis reactions on the nanowire tips. As a result of the hydrolysis, Zn(OH)_2_ and H_2_Se are produced, the latter can undergo oxidation with oxygen from the purged air that promoted the reactions of hydrolysis.

In the next section, the main processes and most recent advances in the field of shape-tailored nanostructure synthesis by PLAL are discussed.

### 2 Current status of the shape-controlled synthesis of nonspherical NPs by PLAL

**Figure 4:**
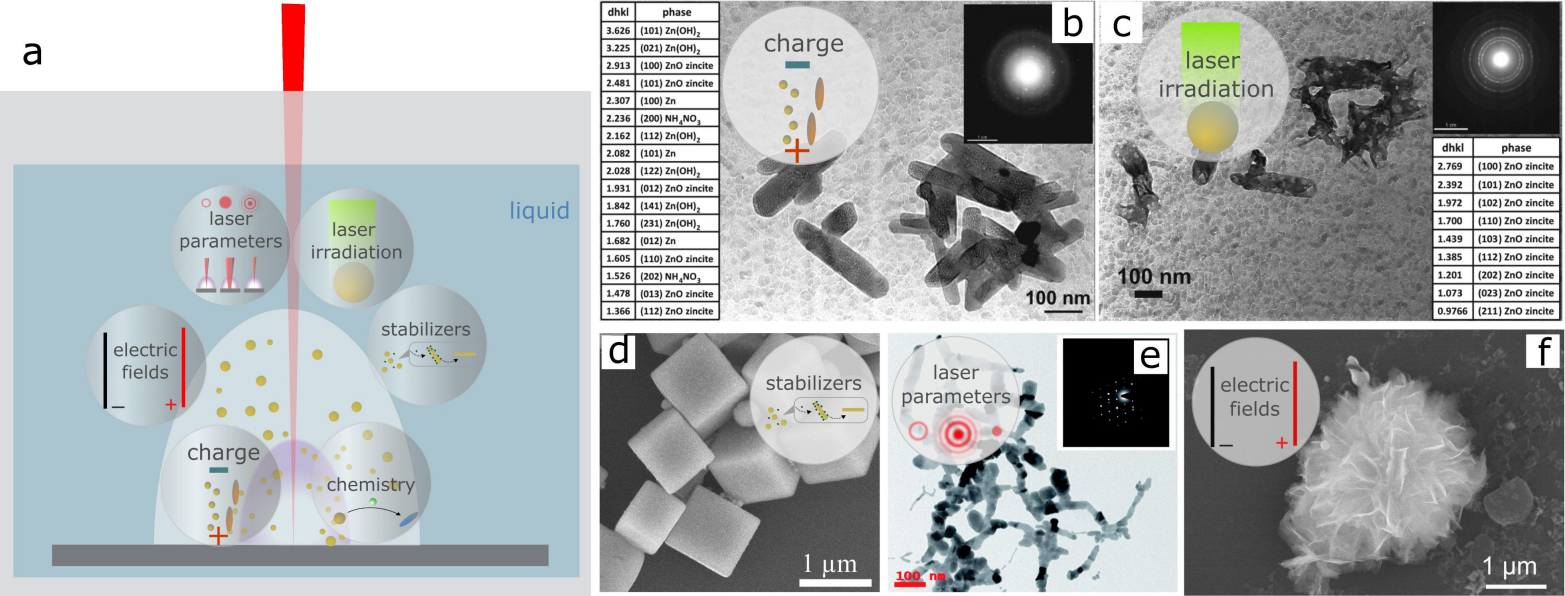
Key parameters for shape-controlled synthesis of anisotropic nanomaterials by laser ablation in liquids: a) the scheme illustrating major parameters influencing NP shape to enable controlled synthesis, b)–f) –examples of nonspherical NPs prepared by laser ablation in liquids: b), c) TEM image of ZnO:N nanorods prepared by laser ablation in an ammonium nitrate solution before (b) and after laser modification (c). Figure 4b,c was reprinted from [2], *Nano-Structures & Nano-Objects*, Vol. 12, by N. Tarasenka; A. Butsen; V. Pankov; T. Velusamy; D. Mariotti; N. Tarasenko, “Laser assisted preparation of doped ZnO nanocrystals“, Pages 210-219, Copyright (2017), with permission from Elsevier. This content is not subject to CC BY 4.0. d) SEM image of silver oxide nanocubes formed by laser ablation in the solution of polysorbate 80. Figure 4d was reprinted with permission from [3]. Copyright 2011 American Chemical Society. This content is not subject to CC BY 4.0. e) TEM image of Ag nanoribbons, formed by ps laser ablation using cylindrical focussing geometry. Figure 4e was reproduced from [4] ("Silver nanoribbons achieved by picosecond ablation using cylindrical focusing and SERS-based trace detection of TNT", © 2020 H. Marrapu et al., published by The Royal Society of Chemistry, distributed under the terms of the Creative Commons Attribution-NonCommercial 3.0 Unported Licence. https://creativecommons.org/licenses/by-nc/3.0/). This content is not subject to CC BY 4.0. f) ZnO nanoflower prepared by laser ablation in water in external electric field.

In principle, the conditions at each stage of the ablation process might be changed to adjust the morphology of NPs. The attractive feature of the laser ablation technique in liquid is an existence of a number of experimental parameters that can be used to fine tune and tailor the NP parameters and properties (Figure 4a). Although controlled NP formation is promising, the influence of individual parameters remains unknown due to the complexity of the processes involved. As a result, a mixture of NPs with different morphologies and sizes is often produced. Since laser ablation in liquid typically does not imply usage of any surfactants or stabilizers, the produced bare NPs can spontaneously assemble and grow into nanostructures of different shapes. The latter processes often occur in solution after synthesis and may significantly change the shape of the formed NPs compared to that of the as-formed structures. For example, Solati et al. in [32] studied the effects of laser pulse energy and wavelength on the ZnO NP characteristics, and observed the formation of a mixture of nanosheets and spherical NPs for the ablation using the 1064 nm laser wavelength, while at 532 nm only spherical NPs were observed. The authors observed nanosheets only in scanning electron microscopy, while transmission electron microscopy showed only spherical nanostructures for both wavelengths used. Therefore, the authors attribute the formation of sheet-shaped particles to the adhesion of the spherical NPs. The increase of the laser pulse energy favoured the formation of spherical particles and hindered the growth of sheet-shaped structures, which the authors explained by the decrease of the adhesion of NPs. The variation of the NP shape can be more significant in organic liquids. For example, Singh et al. [33] produced drop-shaped ZnO quantum dots by laser ablation of Zn in methanol, which were prone to spontaneous self-assembly after the end of the synthesis process with the formation of dendritic structures. The authors discussed the evolution of NP shape based on the electrostatic attraction between species released from the plasma plume and liquid molecules, so that the resulting shape of the forming NPs would depend on the dielectric constant and dipole moment of the liquid used for ablation. These parameters of the liquid would have an impact on surface charges that determine the processes of assembly and arrangement of the formed material into specific geometries and architectures. For the drop-shaped NPs, observed in [33], a dipole moment in the NPs is induced due to the difference of charge density at the sharper end of the structure. As a result, the assembly of the droplet-like shapes form organized dendritic structures instead of loose aggregates due to a stronger repulsion by the sharp ends of the nanodroplets as compared to that of the wider parts. The architecture of the formed dendrites depends on the spatial organisation of the interacting nanodroplets as well as on methanol molecules, which are arranged in a way to reduce overall electrostatic energy. Therefore, organic liquids favour not only size quenching of the NPs during synthesis, but also transformation of the NP shapes during ageing and storage.

According to the described mechanism, the induction of nonspherical shapes is possible by either variation of the conditions of plasma propagation and expansion or by manipulation of the growth and assembly of the formed NP seed to induce their anisotropic growth and assembly. Therefore, variations in parameters such as liquid composition (as, for example, in Figure 4b–d) and laser focusing conditions (Figure 4e) can create favourable conditions for the formation and growth of anisotropic nanoparticles. Another approach is based on the introduction of external electric, magnetic, or temperature fields during the ablation process (Figure 4f). In the following sections, the strategies for the shape-controlled synthesis of nanomaterials by laser ablation in liquids are summarized.

### 3 Discussion: Strategies for NP shape manipulation in PLAL synthesis

#### 3.1 Control of the liquid composition and parameters

One of the versatile routes towards shape-controlled laser-assisted synthesis is the variation of the liquid parameters, including solution composition, pH values, and temperature. The most commonly used strategies are based either on the ablation in organic liquids or on the addition of surfactants or ionic species which participate in the processes of NP nucleation and growth and alter the structure and overall morphology. Although this approach has several limitations and may affect colloid purity, varying the liquid composition offers a simple and convenient way to manipulate nanoparticles during and after synthesis. This variation can alter the mechanisms governing not only nanoparticle formation but also their growth and assembly, leading to novel architectures and hierarchical structures.

**Figure 5:**
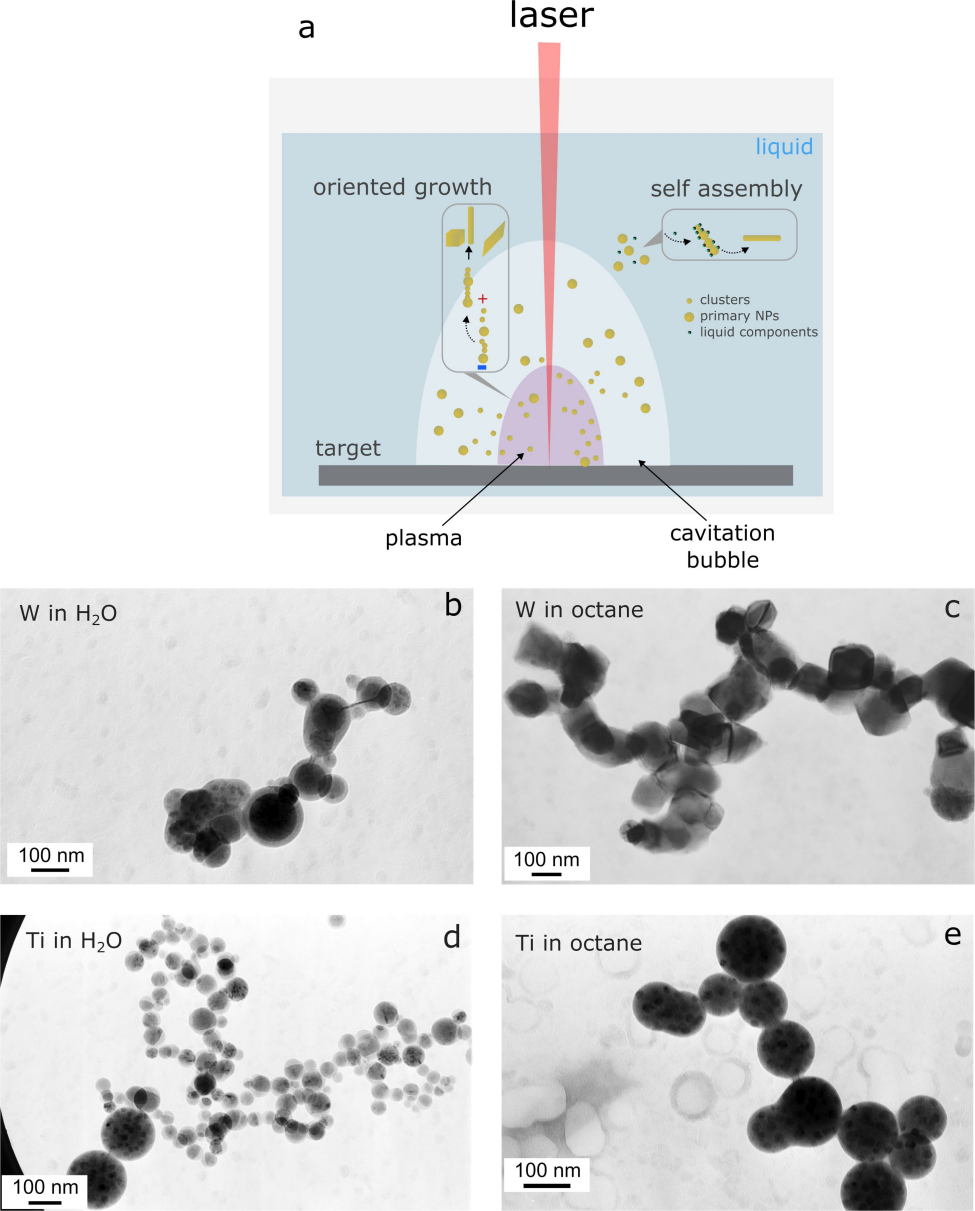
Demonstration of the influence of liquid composition on the shape of prepared nanomaterials: a) scheme demonstrating the processes of oriented growth and self-assembly of the nanomaterials into the anisotropic nanostructures, b)–e) TEM images of the NPs prepared by ns laser ablation of W (b, c) and Ti (d, e) in H_2_O (b, d) and octane (c, e) showing the dependence of the morphology on the composition of both liquid and metal target.

The influence of the liquid composition on the shape of the resulting NPs is demonstrated in Figure 5, where the TEM images of the NPs produced by Nd:YAG (1064 nm) nanosecond laser ablation of W and Ti targets in different solvents are presented. For both metals, the ablation in water produces spherical NPs, while in octane, the W ablation results in predominance of cubic shapes. However, the ablation of Ti in similar conditions in octane still only results in spherical NPs, which can be attributed to the difference of physicochemical processes occurring in both cases. These results demonstrate that the mechanisms and underlying processes of the nanomaterials shape change are dependent on both the material of the NPs and composition of the liquid or added molecules. However, as discussed above, the major challenge is still the lack of the knowledge of the mechanisms involved, which hinders the development of the exact methodology of NP production with an exact shape.

**Figure 6:**
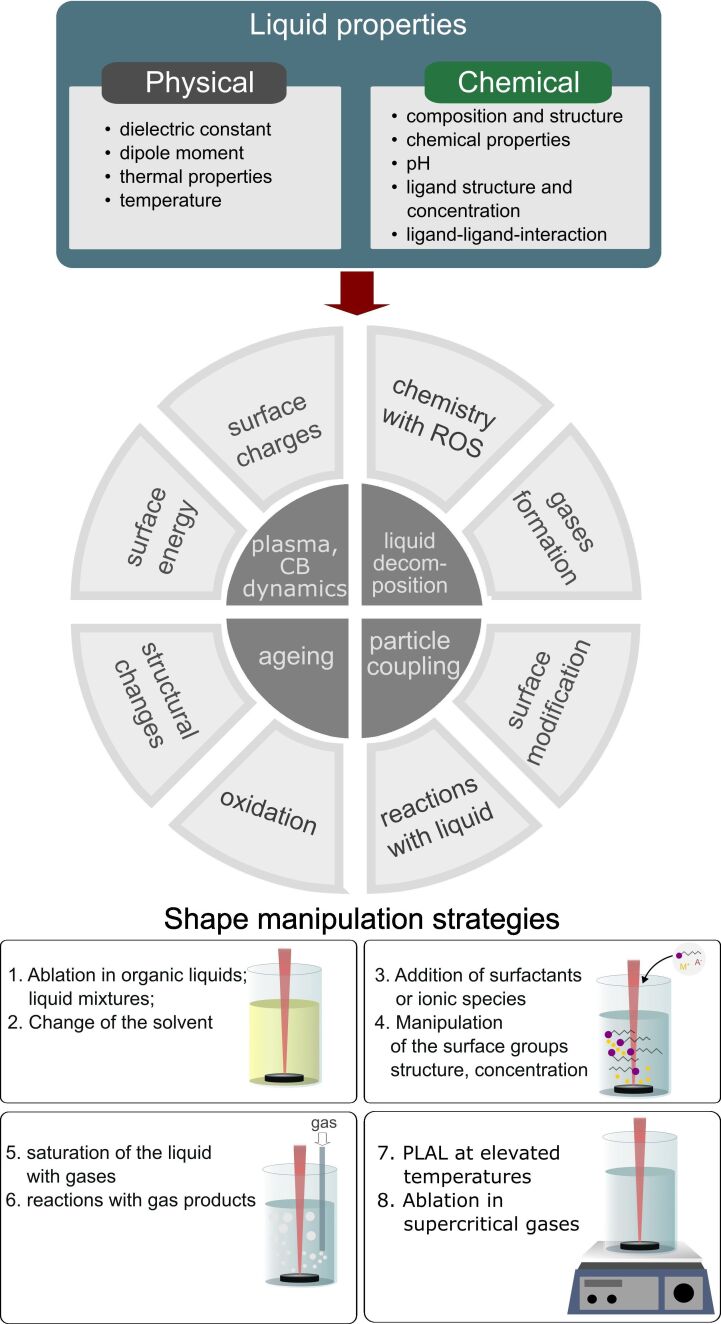
A scheme illustrating the impact of liquid properties on NP formation processes and main shape manipulation strategies during PLAL based on change of liquid properties.

In fact, a change of the liquid composition alters all the conditions of the plasma and cavitation generation and evolution, which explains the shape change (Figure 6). The composition of the liquid also guides the chemical processes occurring with the ejected species both in the cavitation bubble and in a liquid. As a result, shape change can occur as a result of chemical processes, structural transformations, or phase transitions occurring in NPs. For example, in [34–35], laser ablation of Bi has been shown to preferentially produce spherical Bi NPs in acetone. However, in water, spherical NPs were initially formed and further transformed into nanoflakes and nanosheets which were composed of Bi subcarbonates (BiO)_2_CO_3_ and (BiO)_4_CO_3_(OH)_2_. Furthermore, a rapid change from water to acetone prevented the formation of nanoflakes, while the addition of even 1% of water to acetone resulted in the formation of nonspherical nanostructures.

If stabilizers are added to tune the NP shape, the observed transformations are usually related to the later stages when the NPs, being usually spherical upon release from the cavitation bubble, are transferred into a liquid. Such seed NPs are typically having well defined crystalline structure, surface composition, and specific surface free energy. The assembly and growth of the NPs is explained by the decrease in surface energy. If a liquid contains surfactant molecules or ionic species, their selective adsorption at specific facets of seed NPs can be expected to be governed by the decrease of surface free energy [36–37]. The density and binding energy of the ligands can significantly differ for different crystal planes. As a result of specific adsorption, the crystal facets with adsorbed ions become blocked, which promotes an oriented attachment and growth only in a specific direction. This strategy for shape manipulation was used in the works of Liang et al. [38], who produced Zn(OH)_2_ nanosheets by laser ablation of Zn in a sodium dodecyl sulphate solution, while in water similar conditions resulted in preferentially spherical NPs. Furthermore, the authors showed the approach to tune the interlayer spacing in Zn(OH)_2_ nanosheets, which was depended on the chain length of the organic stabilizer. Another example of shape-controlled synthesis in the presence of organic stabilizers is the formation of silver nanowires in a process of fs laser ablation of a silver target in solution of sodium citrate and polyvinylpyrrolidone in water shown in [39]. The citrate ions selectively adsorbed at the (111) facets of the initially formed spherical seed NPs, so that the following growth occurred only within (111) plane.

Manipulation of the structure and concentration of surface groups influence coupling between the interacting nanocrystals and open the pathways for engineering nanostructure shape. As demonstrated in [40], the AgBr nanosheets were only formed if the CTAB concentration exceeds 0.001 M, while at lower concentration the growth of spherical NPs is favourable. As a mechanism, the authors suggest the coordination of the interaction of Ag species, ejected from the target with Br ions from the solution by the CTAB stabilizer molecules. Similar action during NP growth can be expected upon addition of some metal ions (Ag^+^, Fe^3+^, Co^+^) and complexes (W(CO)_6_) [41]. These additives are known to promote growth of nonspherical shapes in colloidal chemical synthesis by preferential adsorption on specific crystalline planes.

Another example of the influence of ligand concentration on nanoparticle shape is given in [42], where high ligand density led to truncated octahedral PbSe nanostructures, while reduced surface ligand concentration (achieved by washing) resulted in cubic structures. This result shows that addition or removal of surface ligands may result in nanocrystals of different shapes. The length of organic chains in stabilizing ligands influences particle–particle coupling; therefore, the exchange of stabilizing agents will influence the overall shape of nanomaterials [43–44].

Further surface engineering by the control of ligand–ligand interactions can result in the formation of novel nanoarchitectures and superlattice structures [45]. This can be achieved, for example, by varying the temperature of the surrounding liquid. The dynamics and parameters of the plasma and cavitation bubble are also temperature dependent. The demonstration of the influence of the liquid temperature was provided in a number of studies. In [46], ZnO and Zn(OH)_2_ of different sizes and morphologies were formed by laser ablation in a liquid environment of different temperatures (50, 70, and 90 °C). The temperature increase enhances the interactions of a liquid with the formed NPs, thus promoting their surface modification and oxidation, and influencing the processes of ageing, oxidation, aggregation, and agglomeration. As a result, in [36], the increase of the ambient temperature was demonstrated to induce oriented growth of the NPs with the formation of nanospindles, whereas at room temperature only isotropic enlarged aggregates were observed. The changes in the structure were observed even at slightly elevated temperatures (35 °C), which indicates high sensitivity of the growth mechanisms to temperature increase. A similar effect has been shown by Haram et al. in [47], who observed formation of Ag and Au nanochains, nanorings, and superclusters as a result of laser ablation in distilled water at elevated temperatures (70 °C). The nonspherical shape was attributed to the fusion of NPs under elevated temperatures which facilitates the formation of nanochains and nanoclusters.

A different strategy based on the utilization of fluid and superfluid noble gases is shown in [48]. Such liquid media are characterized by quantized one-dimensional vortices, which exhibit steep pressure gradients near their cores and can therefore trap ablated atoms, clusters, and primary NPs. Once the NPs are trapped, the directions of their growth are largely restricted to agglomeration only along the vortex direction. As a result, coalescence of the atoms and clusters in the vortices results in the production of filament-like nanostructures and growth of nanowires with length up to several centimetres [48]. This approach has shown to be quite versatile and applied to the nanosecond laser synthesis of nanowires of various metals including noble (Au [48–49], Cu [49]), alkali, and alkali-earth metals (Rb [49], Cs [49], Ba [49]) as well as refractory metals (Nb [50], W [50], Re [50] and Mo [50]).

Upon interaction with a high-temperature plasma plume, liquid molecules undergo decomposition with the production of reactive species or gaseous products, which can be considered as another strategy for the shape control of growing NPs. The exact composition of the produced species is dependent on the composition and pH of the solution, but typically highly active species, such as radicals and ions, are formed. Their adsorption and reactions at the NP surface results in composition, shape, and structure transformations induced in NPs. As an example, Zhang et al. [30] explained the formation of Mn-doped Ni(OH)_2_ nanosheets, observed during Mn target ablation in a nickel chloride solution, by the involvement of hydrogen radicals, hydroxide ions, and hydroxyl groups reacting with Ni^2+^ ions. The authors state further shape transformation of the formed nanosheets into hierarchical spheres. In a similar process, Hunter et al. [51] prepared nanosheets of mixed nickel and iron hydroxide with a complex composition ([Ni_1−_*_x_*Fe*_x_*(OH)_2_](NO_3_)*_y_*(OH)*_x_*_−_*_y_*·*n*H_2_O). The authors used nanosecond ablation of the dispersed metal powders in a salt solution of the second element. The effect of hydrogen ions formed as a result of a liquid decomposition was also studied in [52] for the process of MoO_3−_*_x_* nanosheets formed by fs laser ablation of MoS_2_ powders suspended in liquid. The authors used water/ethanol mixtures to get insights into the influence of a liquid media on the underlying processes, and came to the conclusion that molybdenium oxide nanosheets could only be formed upon an ethanol concentration in the range of 80–95%. In ambient water, the formation of nanobelts occurred. The explanation of the observed regularities is based on the processes of the initial MoS_2_ oxidation that started from H*_x_*MoO_3_ NPs, containing the surface –OH_2_ terminations that the authors attribute to H_3_^+^ ions generated as a result of laser-induced ethanol decomposition. However, increasing the ablation time also induced the transformation of nanosheets to nanobelts. Different processes, however, were observed by Zuo et al. [53], who reported the formation of MoS_2_ nanosheets of elliptical shape as a result of fs laser ablation of a MoS_2_ target in FeCl_3_ solution. Despite the ablation in an aqueous solution, nanosheets preserved the MoS_2_ composition. Electron diffraction results reported in the cited work revealed the formation of an FeS_2_ phase, suggesting that Fe ions adsorb onto the active planes of the MoS₂ target during exfoliation, thereby inducing changes in phase and morphology.

The role of hydrogen peroxide in the preparation of nanosheets by laser ablation was further studied by Azadi et al. [54], who performed laser ablation of Cu in a hydrogen peroxide solution by ns laser pulses. As a result, the dependence of the CuO content in the nanosheets on hydrogen peroxide concentration in solution was found, and the conclusion on the CuO nanosheet formation via reactions of the ablated Cu with H_2_O_2_ was made.

Other products of liquid decomposition are gases, produced either through liquid evaporation or from reactive species generated during decomposition, such as hydrogen, hydrogen peroxide, and oxygen. The type of gas emitted depends on the liquid composition and can influence the morphology of the resulting NPs. As shown in [55], the ablation in ethanol–water mixtures provides a higher yield of gaseous products which favours the formation of hollow nanospheres. This effect observed in water–ethanol mixtures is explained by a longer cavitation bubble lifetime as compared to that of pure water [56–57]. Similarly, saturation of a liquid with gases also provides a route to synthesize hollow nanospheres, as shown in [58]. The reactive gases, such as H_2_, O_2_, and CO_2_ can also direct the growth of nonspherical NPs by adsorption on the seed NP surface and block the growth along specific directions [59–60]. The ambient gases can also induce exfoliation of layered materials, resulting in 2D nanostructures. This approach is utilized for the formation of carbon-based and metal dichalcogenide nanosheets. Apart from gases, ions and molecules present in the solution can participate in the exfoliation as well as applied ultrasound. The application of ultrasound can be useful to control the pressure in laser-generated cavitation bubbles. In [61] ultrasound application during PLAL allowed the formation of bismuth nanosheets, while bismuth NPs formed without ultrasound action in the same experimental conditions of ns PLAL of a bismuth target in water. The pressure waves induced by ultrasonication influence the cavitation bubbles, formed by laser ablation, by creating regions with high and low pressure. As a result, bismuth species are allowed to nucleate and grow in the regions of low pressure, which promotes planar shape of the forming material.

#### 3.2 Laser-induced modification of pre-formed NPs for shape change

Another strategy for shape manipulation can be based on utilization of laser-assisted processes in multistep synthesis approaches. In these methods, the laser ablation of a solid target is used to prepare initial colloids which are further processed by unfocused laser irradiation as a second step. This strategy has developed into several methods usually categorized as laser-induced fragmentation, laser-induced melting, or laser-induced modification. Such multistep processes open up ways for precise manipulation and fine tuning of NP parameters through self-assembly, seed-mediated growth, re-shaping, or chemical interactions with the colloid medium [62–63]. The exact mechanisms and resulting structures are determined by a number of experimental parameters (laser fluence, laser pulse duration, exposure time, laser wavelength, composition and properties of the surrounding liquid and NPs, and kinetics of relaxation processes in the surrounding medium), thus providing a playground for tailoring NP properties.

**Figure 7:**
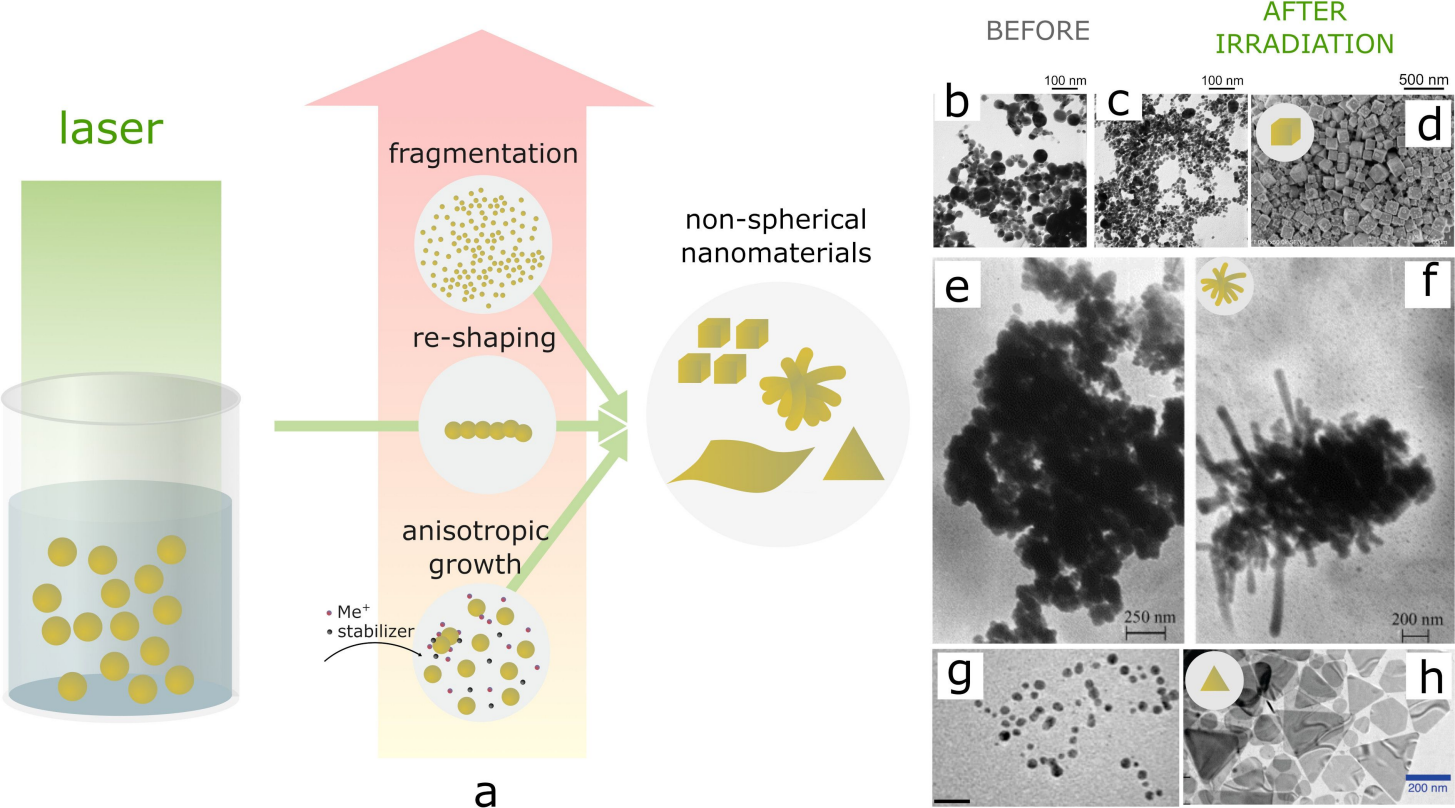
Shape changes induced by laser irradiation of the NPs in colloids: a) scheme demonstrating possible processes with spherical NPs under laser action with different fluences; b)–g) examples of shape changes of NPs induced by laser action: b)–d) TEM images of silver NPs as-prepared in a 25% acetone–water solution (b), after laser fragmentation (c), and SEM image of Ag nanocubes formed after fragmentation and ageing (d). Figure 7b–d was reprinted from [64], *Colloids and Surfaces A: Physicochemical and Engineering Aspects*, Vol. 529, by T. Tsuji; M. Kikuchi; T. Kagawa; H. Adachi; M. Tsuji, “Morphological changes from spherical silver nanoparticles to cubes after laser irradiation in acetone–water solutions via spontaneous atom transportation process”, Pages 33–37, Copyright (2017), with permission from Elsevier. This content is not subject to CC BY 4.0. e),f) TEM micrographs of Ag NPs formed by ns laser ablation before (e) and after (f) 532 nm laser modification. Figure 7e,f was used with permission of Elsevier, from [65], “Laser-induced modification of metal nanoparticles formed by laser ablation technique in liquids”, by N. V. Tarasenko; A. V. Butsen; E. A. Nevar, *Applied Surface Science*, Vol. 247, 1–4, Pages 418–422, Copyright (2005); permission conveyed through Copyright Clearance Center, Inc. This content is not subject to CC BY 4.0. g),h) TEM images of silver NPs seeds before g) and after h) 627 nm LED excitation. Figure 7g and Figure 7h were adapted with permission from [66]. Copyright 2010 American Chemical Society. This content is not subject to CC BY 4.0.

In general, laser pulses of moderate fluences and longer durations are preferable for shape modification, providing mild conditions and sufficient time for thermal processes of heating and melting to occur (Figure 7a,f). These mild conditions favour the heating and melting of NPs instead of their fragmentation, which is required for targeted shape change.

The laser parameters required to melt the particles by laser pulses can be determined by estimation of the absorbed and dissipated heat fluxes from the laser heated NPs. Apart from the laser parameters, these fluxes are determined by NP size and composition as well as by properties of the surrounding liquid which define the kinetics of relaxation processes. The balance between absorbed and dissipated energy is usually considered [63,67–70]:


[3] $$E_{\text{abs}}=E_{\text{melt}}^{\text{th}}+E_{\text{melt}}+E_{\text{boil}}^{\text{th}},$$

where *E*_abs_ is the energy absorbed by the NP, *E*_melt_^th^ is the thermal energy required to reach the melting point *T*_m_, *E*_melt_ represents the melting energy, and *E*_boil_^th^ represents the thermal energy required to heat the system from the melting point to the boiling point *T*_b_. These energies are calculated using the formulae (Equations 4–7):


[4] $$E_{\text{abs}}=\sigma_{\text{abs}}^{\lambda }\Phi t_{\text{p}},$$

[5] $$E_{\text{melt}}^{\text{th}}=mC_{\text{p}}^{\text{sol}}\Delta_{T_{0}}^{T_{\text{m}}}T,$$

[6] $$E_{\text{melt}}=mL_{\text{m}},$$

[7] $$E_{\text{boil}}^{\text{th}}=mC_{\text{p}}^{\text{liq}}\Delta_{T_{\text{m}}}^{T_{\text{b}}}T.$$

In the equations above, σ^λ^_abs_ is the absorption cross section at the wavelength λ, Φ is the laser irradiance (W/m^2^), *t*_p_ is the pulse duration, *m* is the mass, *C*_p_^sol^ and *C*_p_^liq^ are the heat capacity of the NPs in solid and liquid state, respectively, and *L*_m_ is the latent heat of fusion.

These equations allow to determine the threshold irradiance required to melt NPs of specific sizes and compositions using laser pulses with a selected duration, fluence, and wavelength:


[8] $$\Phi_{\text{melt}}=\frac{E_{\text{melt}}^{\text{th}}+E_{\text{melt}}}{\sigma_{\text{abs}}^{\lambda }t_{\text{p}}}.$$

The dissipation of the absorbed energy occurs via two mechanisms, convective and radiative. Typically, radiative fluxes are several orders of magnitude lower than convective ones; thus, it is usually excluded from consideration and only convective fluxes φ_conv_ are estimated. This can be done using Equations 9–10:


[9] $$\varphi_{\text{conv}}\left(r,x\right)=4\pi r^{2}h\left[T_{\text{NP}}\left(r,x\right)-T_{\text{b}}^{\text{l}}\right],$$

[10] $$h=\frac{\text{Nu }k}{L},$$

where, *h* is the heat transfer coefficient, Nu is the Nusselt number, *k* is thermal conductivity of the surrounding liquid, *L* is the characteristic length, and *T*_b_^l^ is the surrounding liquid boiling temperature.

In order to analyse the reached temperatures and occurring thermal processes, the dissipated heat fluxes should be compared with the input flux due to laser irradiation:


[11] $$\varphi_{\text{input}}\left(r,x\right)=\sigma_{\text{abs}}^{\lambda }\left(r,x\right)\Phi .$$

In the equations above, several quantities (irradiance, melting temperature, absorption cross sections) depend on NPs parameters, such as size (*r*) and composition (*x*). Theoretical description of these dependences can be found elsewhere [67,71–72]. The absorption 
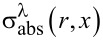
, extinction 
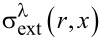
, and scattering 
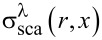
 cross-sections can be calculated using the formulae (Equations 12–15) [67]:


[12] $$\sigma_{\text{abs}}^{\lambda }\left(r,x\right)=\sigma_{\text{ext}}^{\lambda }\left(r,x\right)-\sigma_{\text{sca}}^{\lambda }\left(r,x\right),$$

[13] σextλ=2πK2∑n=1∞(2n+1)Re(an+bn),

[14]

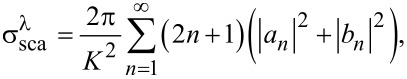



where *n* is the multipole order; *K* = 2π*n*_r_/λ, and *n*_r_ represents the real part of the refractive index.

The melting temperature of the NPs, *T*_m_(*r*,*x*), is also dependent on the size, which can be obtained from Equation 15, derived by Buffat and Borel in [72]:


[15] $$T_{\text{m}}\left(r,x\right)=T_{\text{m}}^{\text{bulk}}\left(x\right)\left\{1-\frac{2}{r\rho_{\text{s}}\left(x\right)L_{\text{m}}\left(x\right)}\left[\sigma_{\text{s}}\left(x\right)-\sigma_{\text{l}}\left(x\right){\left(\frac{\rho_{\text{s}}\left(x\right)}{\rho_{\text{l}}\left(x\right)}\right)}^{2/3}\right]\right\}.$$

In Equation 15, 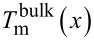 represents the melting temperature of the bulk material, which depends on its composition; ρ_s_ and ρ_l_ are the densities of the solid and liquid materials, and σ_s_ and σ_l_ are the surface energies of the solid and liquid materials.

The size of the NPs strongly influences the input and dissipative fluxes, as it follows from the equations above. Moreover, the dissipated heat may be sufficient to evaporate the surrounding liquid, creating a gaseous envelope around the nanoparticles that should be taken into account. [73]. The presence of the gaseous bubble around the heated particles influences the kinetics of the relaxation processes as well as the absorption of the laser beam by the NPs. In [67], the conditions required for melting silver and copper NPs with the formation of a metastable AgCu alloy were analysed in detail, and it was estimated that for small (*r* ≤ 2.8 nm) and relatively large NPs (*r* ≥ 390 nm for pure copper and *r* ≥ 75 nm for pure silver) the melting and alloying does not occur at the used laser irradiance (3 × 10^11^ W/m^2^). This is due to large energy dissipative losses for small NPs or insufficient laser energy for large ones. For the particles of other diameters, melting is shown to occur; however, the resulting processes depend on the formation of a gaseous envelope as well as the kinetics of melting, relaxation, and liquid evaporation [67].

The approach based on melting and reshaping of NPs using ns, ms, and CW lasers has been applied in a number of works. Among the first, ns laser-induced shape modification of spherical silver NPs to nanowires in colloids has been demonstrated by Tsuji et al. [74] and Tarasenko et al. [65] (Figure 7e,f). The diameter of the formed nanowires was close to the diameter of the initial spherical NPs, so the suggested mechanism was laser-induced melting and fusion into nanowires. The formation of gold nanowires from gold NPs was further observed by several research groups [75–76]. Similar transformation of spherical silver NPs into one-dimensional nanostructures under laser irradiation was further demonstrated in other liquid media [77].

Some works report shape transformations by using high enough fluences to enable fragmentation as well as too low fluences for particles melting. Tsuji et al. [64] used fragmentation of pristine silver NPs in acetone–water solution to induce further re-shaping into cubic nanostructures (Figure 7b–d). The formation of the nanocubes occurred upon ageing of the colloid during several days, and the authors emphasize the importance of the laser fragmentation step in this process.

If the laser fluence is too low to melt the particles, the shape transformation can be induced using photochemical processes. However, in this case, the addition of precursors, reducing, and stabilizing agents is required. The pristine NPs that are present in the solution act as seeds, while photoinduced reduction occurs at their surface followed by anisotropic growth. As a demonstration of this approach, Maillard et al. [78] reported the transformation of spherical silver NPs to triangular or disk-shaped nanostructures by CW laser irradiation of Ag seeds mixed with AgNO_3_ and citrate in solution. The authors demonstrate the photoreduction processes guiding the growth of NPs while the final shape of the nanostructures was determined by the laser wavelength, since the silver NP absorption spectrum depends on shape. The shape dependence on the laser wavelength in a similar approach was also shown in [66], where re-shaping of silver NPs under LED irradiation with wavelengths in the range of 405–720 nm resulted in dodecahedral, plate-, or rod-like nanostructures depending on the wavelength (Figure 7g,h). In principle, the introduction of any other nanoscale inclusions or powders into the liquid would act in a similar way to the NPs discussed above [79]. This concept was introduced in the works of the A. Manshina group, who developed a remarkably different method of NP production at the target/liquid interface, where the continuous wave laser reduces the precursors at the substrate surface, resulting in the formation and deposition of nanostructures. The method was successfully applied for the formation of nanoflakes [80], nanoflowers [81], and nanofibers [82–83]. In this method, the choice of the organic precursor influences the shape of the produced nanostructures. For example, the formation of Ag nanofibers has been shown to occur via anisotropic growth, using the fibrous products of the organic precursor decomposition for self-templating, which guides the following processes of reduction of silver ions at their surface. The concentration of the organic precursor further influences the shape parameters of the produced nanomaterials [80–81].

Despite usage of long laser pulses is much more widespread for NPs re-shaping, some works report the transformation of shape using ultrashort pulses as well. In the latter case, fs laser action is usually inducing photochemical reactions with liquid media thus resulting in gathering and assembly of NPs in an anisotropic shape. This approach has been used, for example in [84], where the initial spherical NPs were assembled into nanorods using the threads resulting from laser-induced decomposition of cucurbit[7]uril molecules. Alternatively, the formation of one-dimensional nanostructures during laser processing of colloids can occur via linear growth. This mechanism was described in a number of works for various materials. In [85–86] the transformation of copper nanoflakes into nanowires dispersed in ethanol or methanol has been achieved by using femtosecond laser irradiation. As in the case of laser ablation of solid targets, utilization of femtosecond laser beams for laser modification of pre-formed nanomaterials is strongly dependent on the polarization of the laser beam. In [85] copper nanoflakes transformed into nanowires under fs laser irradiation with linear polarization during 1–5 min; however, further laser treatment converted the nanowires to NPs. The authors explain the observed changes by the initiation of nanoflakes ablation under laser action, producing atoms, ions, and nanoclusters, which nucleate into seed NPs, deposited on the surface of the nanoflakes. These NPs act as seeds in further growth processes by attaching the ablated material from the surrounding liquid to first form nanorods and eventually nanowires. The linear morphology in the conditions used is explained by the growth of periodic surface structures perpendicular to the laser light polarization. When the nanoflakes are fully ablated and no longer supply material, nanowire growth stops. At this stage, laser-induced fragmentation takes over, producing the nanoparticles observed in the later stages of laser modification. Several important observations were provided in [85–86], giving an insight on the dependences of the resulting morphology on the laser intensity and solvent used. The number of laser pulses determined the length and diameter of nanowires. For testing of the impact of the surrounding liquid, the process was held in ethanol, methanol, and acetone. In the latter, no nanowires were observed in contrast to the alcohol solutions.

In principle, further shape control strategies in laser-induced modification can be similar to that of laser ablation of a bulk solid target in liquid. For example, to add controllability to the laser modification process, additional stabilizers can be added to the solution during laser-induced modification of NPs. Such an approach allowed Sebastian et al. [87] to transform silver nanospheres to nanowires by nanosecond laser irradiation in a polyvinyl pyrrolidone (PVP) solution in ethanol. The authors underline the role of PVP in shape transformation which occurs via ripening. The preferential interaction of the stabilizer molecules with the {100} facets of nanowires as compared to that with the {111} facets, enabled nanowire growth in the ⟨111⟩ direction. The absorption of NPs is known to be sensitive to the wavelength. Therefore, the variation of the incident laser beam wavelength can be used to direct the process of modification either to the pathway of size modification or re-shaping route. In [76] the formation of Au nanowires was only observed at laser wavelengths of 355 and 532 nm, while a 1064 nm laser beam did not induce shape transformation.

Furthermore, external electric fields can also be applied during laser irradiation of NPs, thus adding to the possibilities of charge manipulation and shape-controlled synthesis. If the excess electrons on the particles surface appear, they might disturb the equilibrium charge distribution at the particle surface and result in dipole nanoclusters, which would linearly grow by attaching NPs from the colloid. As demonstrated by Serkov et al. [88], such excessive charges can result from the applied electric field, decomposition of the stabilizing agent, or thermionic emission from the irradiated NPs. The application of an electric field was also observed to cause elongation of the carbon flakes produced by laser synthesis at the substrate/solution interface in [81].

It is noteworthy that not only additional laser irradiation is applied for the tailoring of the NP shape after laser ablation synthesis. The combined multistep approaches continue to attract the attention of researches by providing control during synthesis. For example, Hu et al. in [89] developed a combined laser-hydrothermal approach for the synthesis of Zn_2_GeO_4_ nanowires based on sequential processes of simultaneous laser ablation of Zn and Ge targets in water, followed by hydrothermal growth in an autoclave.

#### 3.3 Electric-field-assisted laser ablation in liquids

The latest research on laser ablation synthesis of NPs allows concluding that an efficient and versatile approach of shape-controlled synthesis can be based on the application of external fields during synthesis. Despite in external fields the overall picture of occurring processes becomes even more complex, the methods utilizing temperature [90–91], magnetic [92–94], and electric fields [11,95–112] are gaining increasing attention. Under external fields, fabrication of anisotropic nanomaterials of completely different morphologies can be achieved without the admixture of spherical nanoparticles, which is a sufficient prerequisite for shape-controlled laser-assisted nanofabrication. Furthermore, laser-induced crystallization has recently been intensively developed allowing the formation of anisotropic shapes under applied fields [113].

Among external fields, electric fields are the most studied towards the size and shape-controlled synthesis and, therefore, will be the primary focus of the next section. The applied electric fields provide a tool to directly manipulate the surface charges of the NPs, allowing the formation of nonspherical nanomaterials in a single step in water. To date, synthesis by electric-field-assisted laser ablation in liquid (EFLAL) has been demonstrated for a range of nanomaterials, including Ag [100], Au [101], Cu [102], Pt [103], Al [104–105], Sn [106], brass [107], Bi_2_O_3_ [108], GeO_2_ [109], ZnO [11,111], ZnO/carbon composites [111], metal molybdates [97,99], and vanadates [98]. However, in a number of EFLAL works, spherical NPs are produced, while the result of an external action is the increase of NP production rates and size reduction. For example, Sapkota et al. [106] showed a decrease in the average size of Sn NPs with an increase in the electric field strength. This is explained by the Rayleigh instability in the formed NPs induced by electron capture in the applied electric field. The influence of the electric field on the ablation process of brass NPs was studied in [107] and an increase of the ablation yield was observed.

Although the formation of nonspherical shapes was the most common feature of EFLAL, it usually required specific values of electric field magnitudes. Otherwise, nonspherical NPs were observed as an admixture to the spherical NPs. For example, Moniri et al. in [103] observed the admixture of rectangular and hexagon particles in addition to spherical NPs as a result of laser ablation of Pt in liquid in electric fields of higher magnitude. At the same time, under higher electric field magnitudes, spherical NPs size decreased, which might indicate structural reorganization occurring in this case resulting in nonspherical NPs. Ismail et al. [108] also underlined the crucial importance of the electric field magnitude on size and shape of synthesized Bi_2_O_3_ NPs, which changed from submicron spheres in lower electric fields (1.5 V·cm^−1^) to irregular-shaped particles in increased electric fields (7.5 V·cm^−1^) [108]. On the contrary, Al-Haddad et al. [101] found that the formation of an admixture of nonspherical NPs such as cubic, triangular, rhombic, or spindle-like occurs in electric fields of moderate magnitude (2–10 V·cm^−1^), while at lower and higher electric field magnitudes, small spherical NPs are dominant. In the recent work by Mehta et al. [110], nonspherical NPs were formed only in the case of an electric field of 100 V·cm^−1^ and 1000 V·cm^−1^, when cubic, hexagon, and pentagon nanostructures were observed. For a 500 V·cm^−1^ electric field, the particles of only spherical shape were formed similar to those synthesized without any external fields. The authors attribute the observed shape changes to the effect of particles charging under the applied electric field. It can be assumed that the magnitude and direction of the electric field promotes the growth of specific crystal planes in the nanocrystal, resulting in different particle shapes.

**Figure 8:**
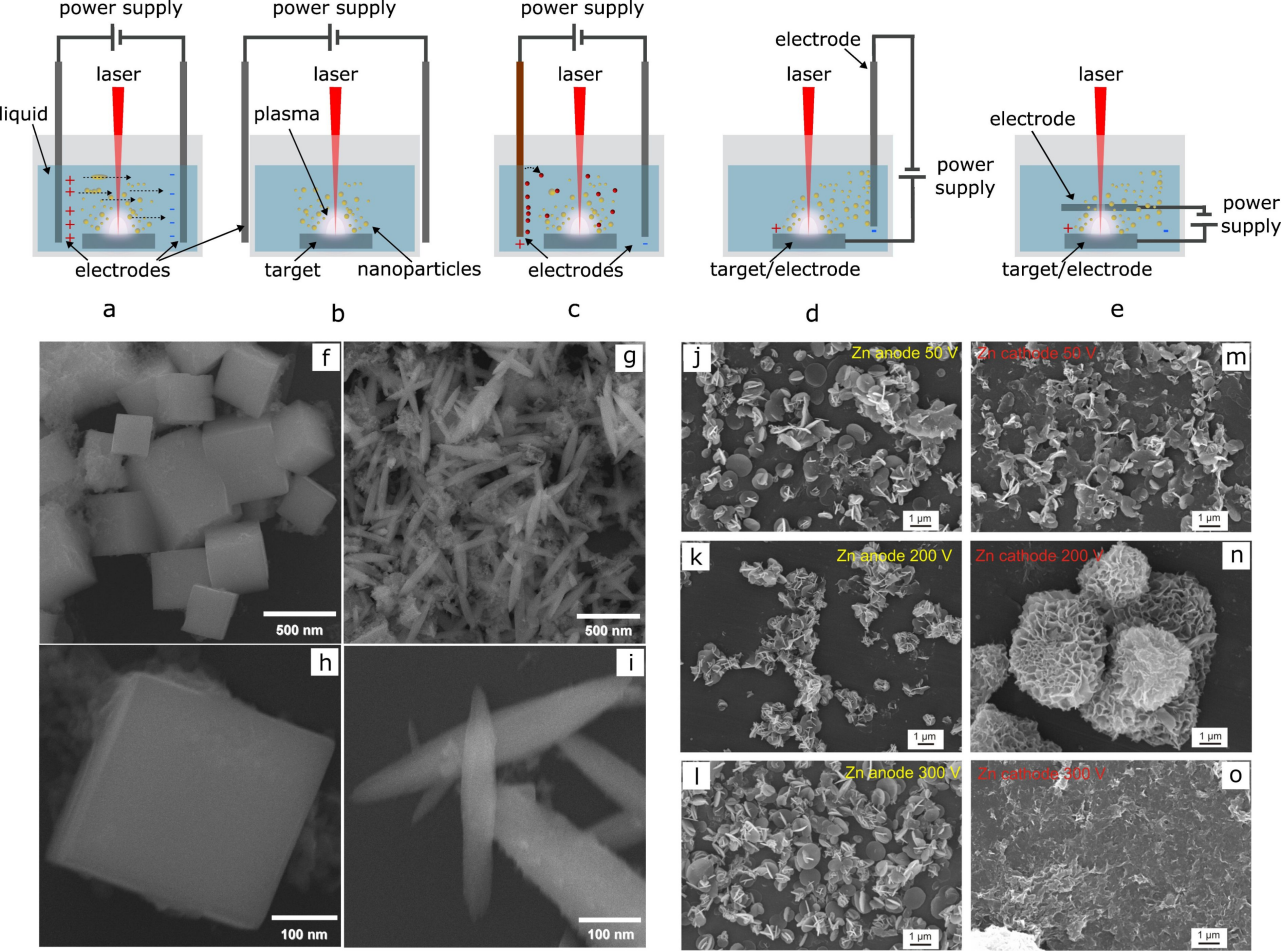
Electric-field-assisted laser ablation in liquids: a)–e) summary of the most commonly used setups for nanomaterials production by EFLAL: a) electric field applied to two parallel electrodes inside the chamber, b) parallel electrodes outside the chamber [110], c) electrochemistry-assisted PLAL with one sacrificial electrode resulting in compound NPs [97–99]; d) the setup with the target connected as the electrode enabling simultaneous deposition of the nanostructures on the counter electrode as used in [11,111]; e) scheme used in [104–105] with an electric field applied parallel to the laser beam; f)–i) GeO_2_ nanostructures formed using EFLAL: (f),(g) low magnification and h),i) high magnification SEM images of micro- and nanocubes and spindles. Figure 8f–i was reprinted with permission from [109]. Copyright 2008 American Chemical Society. This content is not subject to CC BY 4.0. j)–o) ZnO nanoflowers prepared by laser ablation in external electric field depending on the applied voltage and electrode polarity. Figure 8j–o was reprinted with permission from [11]. Copyright 2023 American Chemical Society. This content is not subject to CC BY 4.0.

The complication in interpretation and comparison of the results provided in the literature is also originating from the diversity of the setups used in the EFLAL method, where the configuration of the introduced electrodes might have a role in the synthesis process. In a most commonly used setup, shown in Figure 8a, the voltage is applied to two parallel electrodes immersed in a liquid. In this scheme, species from the ablated target do not directly interact with the electrodes. Instead, the electric field influences the distribution of charged particles in both the plasma and the liquid, guiding their transport towards the electrodes. This setup allows the alteration of the nanomaterials parameters by changing the applied voltage, magnitude, and direction of the electric field. Following the works from the group of G.W. Yang [96,109], who introduced this scheme and method of external fields for the control of shape and structure of NPs, several studies were performed utilizing a similar setup for ablation [102–103,107–108]. In some cases, the electrodes are not immersed in the liquid but are externally applied to the chamber, as for example in [110] (Figure 8b). Several schemes are utilizing electrochemical processes initiated in the applied electric fields. In this case, the application of the voltage to the electrodes induces dissolution of the anode and formation of compound NPs under interaction with the species from the plasma and reactive radicals and ions present in the solution as a result of liquid decomposition (Figure 8c). This approach allowed producing compound nanomaterials, such as metal molybdates [97,99] and vanadates [98] all having nonspherical shapes.

Another variant recently shown in [11,111–112] is the application of voltage directly to the target, while the counter electrode (a conductive inert material) is also introduced into the chamber. In another setup, depicted in Figure 8e, the ablation of the target was performed through a hole in the upper electrode [104–105]. The application of the electrical potential to the target itself offers multiple benefits as it enables simultaneous assembly of the ablated material on the counter electrode with the formation of advanced nanostructures as shown in [11,111,114]. This approach allows to produce in one step functional materials with developed surfaces, which was found to be a beneficial strategy to improve the performance of supercapacitors [11,111] and electrocatalysts [114].

The influence of the electrode geometry on laser ablation under an applied electric field has been studied by Mozaffari et al. in [105]. The study was focused on the evaluation of the efficiency of aluminium target ablation in ethanol under an electric field directed parallel and perpendicular to the laser beam. The authors found that the electric field magnitude and direction determine the laser ablation process by varying the density of seed electrons in a parallel configuration, and by changing the dynamics of the charged species in both configurations. The summary of the setups for electric-field-assisted laser-ablation schemes is presented in Figure 8.

The mechanisms of the underlying physicochemical processes of laser ablation under applied external fields are yet to be developed; however, substantial changes in the occurring processes can be assumed at each stage of the ablation process. This is especially relevant for the processes occurring during the initial stages of laser pulse interaction with a target and plasma evolution. The studies aimed at uncovering the underlying mechanisms and processes at each laser ablation stage continue to appear, providing some insights into distinctive features and differences from conventional processes of laser ablation in liquids. The major parameter in these works is charge and charge distribution, which are largely impacted by an applied electric field. Since ionised and charged species are involved in all processes at each stage of a laser ablation process in liquid, the control of their behaviour can be enabled by the variation of the applied voltage and direction of the applied field. At the initial stage, when the laser beam interacts with the surface, the laser pulse absorption occurs by the electrons from the target. If the target is directly included into the electric circuit, the charging effects of the target surface should also be taken into account (this would be different in the case of laser ablation of cathodes and anodes). The negative polarity of the electrode results in the involvement of additional electrons in the absorption process, thus enhancing the energy transfer from the laser pulse to the target and increasing the efficiency of plasma formation and ablation rate. In the case of laser ablation of a target/anode, the occurring electrochemical processes would induce dissolution of the target and formation of ions that can interact with the components of the plasma and the liquid. As a result, the morphology and structure of the produced NPs would be different from those produced without an electric field (or in case of negative polarity). A significant effect of the initial charge on the target surface on the morphology of the nanostructures has been underlined in the work of Mahdieh et al. [104].

After the laser pulse absorption by the target material, the avalanche ionization results in plasma formation. The laser-induced plasma is partially ionised, thus contains ionised and charged species. That implies that the external electric field would influence the characteristics of such plasma, by changing the charge spatial distribution and dynamics of the charged species. Among the parameters influenced are the ionisation and recombination rates, and thus distribution of charges on the nucleating NP surfaces. The interaction of electric fields with the formed partially ionised plasma plume can also result in charging of the ejected liquid nanodroplets by electron capturing [106]. This effect will result in stabilisation of smaller NPs due to an electrostatic interaction as it is typically observed in EFLAL, but also in induction of electrostatic forces between the NPs, promoting the assembly of asymmetric nanostructures. In addition, the fragmentation of the primary particles due to Rayleigh instability could also influence the size of the NPs. The recombination of ions with electrons occurring in plasma is a competing process to the capture of electrons by NPs and droplets. Therefore, charge control enabled by an external field can be used to control the charging and fine tune the morphology and structure of the nanoparticles. The liquid droplets can also undergo deformation in the applied electric field as a result of its polarization [115]. As a result of the polarization and charge separation, the ejected nanodroplets form an elongated shape. The influence of external electric fields on the charge distribution in plasma and NPs can be expected even in moderate electric fields and is usually dependent on the electric field value [106]. This can also explain the observation of nonspherical nanostructures only at specific electric field values as discussed above.

If laser ablation is performed using relatively long pulses (e.g., nanosecond), at the initial stages the plasma plume is still interacting with the laser pulse. Therefore, the as-formed plasma is subjected to the action of both remaining laser pulse and electrical field. The interaction of the plasma with the laser pulse results in plasma shielding, which usually decreases both the pulse energy reaching the target surface and the ablation efficiency. However, in case of an electric field application, the shielding effect can also be controlled due to the charge separation which depends on the electric field direction. This effect was demonstrated in [105], where charge separation has been shown to reduce the density of charged species in the laser–target interaction zone. As a result, As a result, an increase of the laser energy reaching the target and a decrease of the shielding effect were observed in both parallel and perpendicular electrode configurations. It can be also expected that the charged NPs will be more efficiently removed by the electric field from the beam path, thus decreasing scattering of the laser pulses. Note that the charge separation might also impact the composition of the nanoparticles, especially if the ablation of compound targets is performed. For example, in [114] the possibility of structural manipulation of the MoS*_x_* nanostructures is evaluated by using the dependence of the dynamics and density of the ablated cation and anion species from the applied field.

When an electric field is applied directly to the target, the plasma is expected to have a less homogeneous structure, as found in [11,111]. In [11], the plasma formed during cathode ablation had filamentary structure that, due to a more developed plasma–liquid interface, promoted the formation of nonspherical nanomaterials [11].

The evolution, expansion, and collapse of a plasma plume are also expected to be dependent on the magnitude and direction of the electric field. However, the studies on plasma evolution show different results, which can be related to differences in setups. For example, in [110,116] it is stated that excessive charges increase the density of electrons in the plasma and, therefore, it slows down plasma decay and extends its lifetime. However, the imaging of plasma evolution in [11] showed a lifetime nearly twice as short for plasma generated in external fields of both directions as compared to the that of plasma without an applied external field.

The influence of the electric field on the dynamics and evolution of the cavitation bubble can also be explained based on the charging effects and presence of electrons on NP surfaces. The impact of an applied electric field on the lifetime and pressure in the cavitation bubble has been explored by Mehta et al. in [110]. The cavitation bubble has been shown to have a larger radius and lower pressure as compared to that of laser ablation without any external fields, but was independent of the applied field strength. The evolution of pressure inside the cavitation bubble showed a high pressure (tens of MPa) reached at the initial stages of the bubble oscillation, further bubble expansion with pressure decrease up to the hydrostatic pressure, and finally contracting of the bubble as a result of the action of liquid pressure. This resulted in pressure increase inside the bubble, eventually causing bubble implosion. The collapse of the cavitation bubble also increased the liquid pressure influencing the occurring processes in a liquid, including nucleation and growth of NPs. The presence of charged NPs with excess electrons on their surface in a cavitation bubble induces coulombic interactions, and results in electrostatic pressure [7] directed from the inside of the bubble to its walls. The increase of the electrostatic pressure over the bubble pressure results in earlier release of the NPs to a surrounding liquid and cavitation bubble collapse.

A very recent work by Kharphanbuh et al. [117] used a laser-induced breakdown spectroscopy (LIBS) and a beam-deflection setup (BDS) to get further insight into EFLAL mechanisms. Their results confirm the increase of the bubble size as a result of the electric field impact, which increases the pressure and temperature in the plasma. Authors also admit the perturbation of the electron density and plasma temperature in the electric field, thus altering the interaction region and consequently NP size and parameters. The pressure and temperature inside the cavitation bubble has been shown to increase with the electric field strength. The electric field is also shown to be increased near the bubble wall [118], facilitating perturbation and charge separation in that zone.

As the surrounding liquid also contains ionic and charged species, including the ejected NPs, the applied electric fields would allow manipulation of the directions of the charged species, transport and orientation of the NP growth, as well as assembly after their ejection into the solution. Another effect of the applied electric field would be the influence on the transport of charged NPs to the electrodes. The re-distribution of charge in the solution would also affect the predominant growth mechanism, favouring anisotropic growth and self-assembly of the NPs, which might initially have spherical shape. This effect was observed by Liu et al. [119], who used a two-step approach, where spherical silver NPs were synthesized at the first step followed by the assembly into nanostructures with various shapes, such as nanoflowers, nanoplates, and nanosalts, which occurred after the application of an electric field to the electrodes immersed into a colloid after the end of ablation. The shape was tuned by varying the electric current density. In addition, the morphology was controlled by the liquid change. Li et al. [120] observed the anisotropic NPs only in electric fields of 9.5 V·cm^−1^ if the ablation was performed in ethanol, while in water those were observed independently of the applied field. However, in ethanol, the nonspherical particles had a spindle shape, while EFLAL in water produced nanomaterials of filamentary structure. Similarly, the study of laser ablation of Cu in ethanol in external fields was performed in [102]. The authors observed the restriction of the NP growth in the applied electric fields which resulted in smaller particles in electric fields of higher strength.

Therefore, both physical and chemical processes are impacted by an applied external fields to favour the formation of anisotropic structures, which is a significant pre-requisite towards a shape-controlled NP formation method.

#### 3.4 Shape control by variation of laser parameters and focusing conditions

**3.4.1 Laser parameters:** The laser parameters are the most crucial and therefore most intensively studied experimental conditions which determine all the mechanisms and regularities of NP formation and growth. As a consequence, a large effort in the laser–matter interactions field is directed to the elucidation and optimization of operational parameters of laser systems, such as laser fluence, wavelength, repetition rate, and pulse duration. To date, the obtained knowledge in this direction provides the insight into the routes for controlled and efficient NP production conditions. For example, a recent review by Khairani et al. [121] summarizes the influence of major experimental conditions to reach production rates in the gram scale range. The other direction is the development of size-controlled formation of NPs, as shown in several recent reviews and papers [122]. Some of the examples of major laser parameters that influence the morphology of synthesized NPs are demonstrated in the previous sections. However, since anisotropic nanomaterials synthesis is usually not the major goal of these works, and in many cases, they are formed as admixture to the spherical NPs, nonspherical shape formation mechanisms are often not analysed, and regularities of the nanomaterials shape change are still not well understood.

Among the laser parameters, laser wavelength directly influences the absorption of laser energy by the target material, which has an impact on the penetration depth of a laser beam in a target, on the ablated mass, as well as on photothermal and photochemical processes induced. The difference in photochemistry can be used to control the morphology of the nanomaterials as demonstrated, for example in [123], where laser-synthesized gadolinium oxide nanomaterials had flake-like shape in the case of 532 nm ablation, while nanorods preferentially formed if a 1064 nm laser wavelength was used for ablation of Gd_2_O_3_ in water. The fine tuning of the morphology of the nanostructures has been demonstrated to be achieved by variation of laser fluence [123]. Both Gd_2_O_3_ nanoflakes and nanorods appeared to be more uniform in size and structure if lower fluences were used, while at higher fluences the authors noticed coarser nanostructures attributable to the higher production efficiency, resulting in increased nucleation and growth rates at high fluences. The laser fluence is an important parameter that can be used to manipulate NP formation mechanisms. Thus, understanding its influence is important for the achievement of controlled synthesis of nanomaterials with defined size and shape. At lower fluences, the nucleation and growth of the NPs occurs from the atoms ejected from the target surface via evaporation. At high fluences, melting of a target is another mechanism that is initiated, which gives a fraction of larger particles and a broader size distribution [124]. At the same time, the larger mass of the ejected material at higher fluences provides more primary particles for further nucleation and growth. Another demonstration of the laser fluence on NP shape is the work of Nancy et al. [125], where the shape change of aggregates and chain-like structures has been observed upon laser fluence increase from 5.3 to 47.4 J/cm^2^.

The polarization of laser light is a parameter that has a strong influence on the interaction of the laser pulse with a material and on the surface charges of the NPs. However, this aspect has practically not been addressed in the studies of laser ablation in liquids and need further investigation. To date, the effects of shape-controlled synthesis in the case of laser ablation using different light polarization conditions (linear or circular) have been reported in several articles. Among those, in [126] femtosecond laser ablation of copper microflakes with laser radiation of linear polarization resulted in the formation of Cu nanorods, while laser light of circular polarization produced only spherical nanoparticles. The preferential formation of elongated shapes or one-dimensional structures under application of laser beams of linear polarization were also reported in the case of laser modification of deposited nanoparticles in air [127], glass-imbedded plasmonic nanoparticles [128], and in experiments on laser microstructuring of surfaces, known to be intensively studied for laser-induced surface modification [129]. On the contrary, circular polarization typically produces nanostructures of spherical morphology, as for example in [130], where circular polarization did not influence the shape or size distribution of the nanoparticles formed, while the nanoparticles had spherical shape.

Remarkably, in the above cited studies, shape transformations were induced by ultrashort (femtosecond) laser pulses. Furthermore, low laser fluences are favourable for the observation of polarization-induced effects on the morphology. Ultrashort pulses are relying on multiphoton ionization processes that are sensitive to polarization. For linear polarization, the cross section of a multiphoton ionization is higher than for circular polarization. Furthermore, linear polarization results in asymmetric laser beams which lead to the enhancement of heat flux in the direction of polarization [131]. The use of low fluences allows for the reduction of thermal effects. Thermal processes are less susceptible to the polarization effects, so the morphology changes are related to the laser field variation rather than to thermal effects.

The effect of polarization can be especially significant for plasmonic nanoparticles which are characterized by surface plasmon resonances and, therefore, localized enhancement of the electric field along the direction of light polarization can occur resulting in anisotropic growth of nanorods. Laser-induced shape modification has been observed both in the liquid phase as well as for glass-embedded nanoparticles. In [126], the underlying mechanisms of polarization-dependent shape transformation are discussed, being related to the processes of the multiphoton ionization initiated by fs laser irradiation. The authors show that the surface plasmon polariton waves couple with the incident laser beam wave only in the case of its propagation along the laser light polarization plane. The difference between linear and circular polarization stems from different laser-plasma coupling in both cases.

The change of polarization might guide the growth of NPs produced by laser ablation or influence their assembly during the laser modification stage. In this case, the nonspherical shape is explained by the electric field of the laser pulses which result in the polarization and plasmon–plasmon interactions in metallic NPs. The formation of Ag nanowires in [39] is explained by this effect. The high electric field of the femtosecond laser pulses affect the collective oscillations in the adjacent NPs in a coherent way. This promotes the attraction and attachment of the NPs of the same polarization with the formation of nanowire structures. At the same time, repulsion forces induced through the same mechanism prevent the formation of 2D nanostructures. The length of the formed nanowires has been shown to depend on laser power, so the polarization control can be viewed as another key parameter and tool for NP shape control. A similar mechanism was used to explain the formation of nanoribbons of other materials, including graphene, MoS_2_, WS_2_, and BN in a fs laser ablation synthesis in liquids (water or ethanol) [132]. All these materials were forming one-dimensional nanostructures upon fs laser ablation of powders of corresponding composition in a liquid. Initially, fs lasers initiate fragmentation of the powders with the formation of nanoflakes. Afterwards, those are assembled into one-dimensional nanostructures upon the influence of the electric field of laser pulses. The nanobelts were prone to further modification of their structure due to dangling bonds on the edges and nanostructure surfaces which result in their rolling and curving with the formation of nanotubes.

**3.4.2 Laser focusing conditions:** Another group of often overlooked parameters includes experimental geometry, which implies laser focusing conditions, laser beam profile, target positioning with respect to the laser beam (vertical/horizontal and at focus, above focus, and below focus), liquid layer thickness above the target, and any confinement introduced during the experiment. These parameters can be crucial for the plasma and cavitation bubble propagation, and might significantly vary NP characteristics, including shape.

In a laser ablation experiment, the laser can be focused in three positions: above, at, and below the target surface. The position of the focus is usually adjusted by changing the position of a focusing lens. A change of the focusing conditions results in variation of the laser fluence at the target. Besides, the decomposition processes of the surrounding liquid medium are also affected, which might have an influence on NP shapes and provide a route for shape-controlled NP synthesis. The focusing conditions of the laser beam also influence shockwave generation and propagation, but the related studies are very rare [133]. In [133], the influence of the target position was studied with respect to the spatial and temporal evolution of laser-generated shockwaves. The authors used a beam deflection setup during laser ablation of titanium in water to analyse shockwave propagation dynamics. They observed a significant increase of speed of shockwaves (6.4 km/s vs <3 km/s) and pressure (18 GPa vs ≤1 GPa) in the experiment with a tightly focused beam as compared to that of a defocused beam. If the focal spot was above the target, double shockwaves were generated, the first from the target water interface and the second originated from the in-liquid breakdown. The spatial distribution of the laser energy and pressure is also different, being maximum in the case of in-focus and above-focus conditions, while for below-focus conditions, the shockwave pressure is minimum at the target surface. The deposited laser energy and pressure at the target are another important group of parameters as shown in a number of works [133–135]. Despite these studies are mostly focused on the formation of size dependencies of the NPs on the focusing conditions, the TEM analysis of titanium oxide NPs in [135] clearly shows elongated and rod-like NPs produced in-focus conditions, while mostly spherical NPs of smaller size were produced if the target was placed above the focus. However, Au NPs in [134] show mostly spherical NPs with the admixture of nonspherical shapes for all focusing conditions.

Another way to influence the dynamics of the laser ablation process is the introduction of confinement into the ablation geometry, such as plates in the vicinity of the plasma as demonstrated by Choudhury et al. in [136]. This approach results in the reflection of shockwaves from the introduced walls which compresses the medium adjacent to the plasma plume and induces its heating. These processes influence the thermalisation of plasma, slowing down its cooling. Therefore, synthesis in confined geometries can be used to control NP nucleation and growth processes. Upon fast cooling, the nucleation process dominates over aggregation and growth, thus providing NPs of small size. In a confined geometry, NPs of larger sizes are formed as a result of longer plasma cooling. Another effect from the reflected shockwave is the change of electron density distribution in plasma by inducing perturbations in the medium. In the conditions described in [136], the interactions of the reflected shockwaves cause an increase in the electron charge density in time period of 15–21 μs from the laser pulse action with a rapid decrease after that.

Among the focusing conditions, laser beam shape influence still remains practically unexplored with respect to the NP synthesis in liquids. In a typical laser ablation synthesis experiment, a Gauss beam profile is used. It is characterized by a tight focus in both transverse and propagation axes, and highest power densities in the beam centre. However, nonuniform intensity distribution in the beams of other profiles, such as annular or Bessel, would strongly affect the shape and parameters of the plasma and cavitation bubble, their confinement, propagation and temporal evolution, hydrodynamic trajectory of the ejected target material, as well as pressure relaxation. As a result, the selection of the laser beam profile in a laser ablation experiment can be considered as another tool for shaping the forming nanostructures, similarly to what it is now used for the formation of different topologies on the materials surfaces.

However, for NP production, the beam shapes other than Gauss are still rarely applied. After some preliminary works performed by Menendez-Manjon et al. [137], who used flat-top beam for synthesis of silver NPs in air, and Podagatlapalli et al. [138], where a Bessel beam was utilized for laser ablation of silver in water, there were not many works utilizing non-Gaussian beams in laser ablation production of NPs. Among different beam profiles, Bessel beams gauge the most interest in the material processing field due to unique properties of non-diffracting invariant propagation and self-healing. Therefore, the parameters of the Bessel beam can be beneficial towards controlled formation of nanomaterials of different morphologies. The structure of a Bessel beam, which includes a central lobe and concentric rings, might have an impact on its propagation. The specific energy distribution determines the shape of the plasma and created pressure and temperature gradients, thus strongly influencing the processes of ablation. Due to Bessel beam features, the pressure relaxation is driven in a radial direction resulting in pressure and temperature gradients [139]. This effect has been very recently used for initiation of ejection of the target material and formation of elongated structures at the target surface upon ultrafast Bessel-beam laser ablation of surfaces, as for example in the work by Belloni et al. [140]. The authors show that the radially polarized Bessel beam creates plasma with a cylindrical shape, which creates strong gradients of pressure and temperature allowing ejection of solid material from the bulk and formation of nanopillars. Higher fluences and longer laser pulses favour melting and heating to higher temperatures, producing a material with lower surface tension and viscosity prone to jetting and extrusions [141] impacting the overall morphology. The work underlines a key role of laser energy distribution on the morphology of the produced nanostructures. Depending on laser fluence, three main regimes for surface processing were achieved: translation of a solid target material atop resulting in straight rod-like structures at lowest used fluences, melting and extrusion of viscous liquid material with the formation of slick nanopillars, and liquid jet formation at high fluences resulting in wavy nanopillars. At the highest fluences the jets are consisted of a liquid of low viscosity and characterized by droplet formation and capillary instabilities influencing the overall shape of the structures obtained. Apart from fluence, the mechanism is also strongly dependent on the pulse duration. As a result, longer pulses are shown to favour atomization, extrusion, and jetting of the liquid material, while fs pulses produce nanomaterials using pressure gradients and translation of solid nanostructures. The pulse duration is also influencing the spatial temperature and pressure gradients, reducing them for ps pulses as compared to fs ones. The findings in the work [140] also allows monitoring of the impact of other laser parameters, including laser beam polarization and target positioning with respect to the beam (front or rear side ablation) on the resulting nanostructure morphology. Polarization of a laser beam is influencing the absorption and, therefore, it has been shown that using azimuthal polarization, characterized by decreased absorption, favours the formation of straight nanopillars by the first mechanism.

The outer rings of Bessel beams have a similar energy; however, it is spread over a larger area then that of the central lobe. As discussed in [138], the outer rings might be involved in nonlinear absorption of incident photons by NPs and, moreover, they can act as potential wells, trapping the produced particles. Another possible effect of Bessel beam shape is the generation of multiple filaments in areas processed with outer rings [138,142–146]. The formation of filaments in the processes of laser ablation by Bessel or combined Bessel–Gauss beams represents another tool for generation of nonspherical NPs. As a result, the elongated energy deposition area is created, gaining much attention in materials processing, cutting, and ablation in air, which led to the method of laser-induced filamentous modification [147].

The Bessel beams are usually generated using axicons. The axicons allow tailoring the focal depth by changing the angle of the axicon or incident beam diameter. At the same time, laser power density distribution in a larger area might lead to difficulties in forwarding the applied laser energy exclusively to laser ablation. In [138] the authors admit the simultaneous occurrence of NP formation and fragmentation. However, the experiments in material processing and tissue ablation in medicine have also shown the central lobe to be resistant to distortion upon its propagation in liquid media. This allows a beam to maintain its intensity and deeply propagate even in highly scattering media, which could be a benefit for laser ablation in liquids [148–150]. The created fluence gradients would also be promising for application of Bessel beams in laser-induced modification processes. In [151] the characteristic feature of longer focusing distance of Bessel beams has been used to enable either convex surface structure formation or microparticle formation in liquids depending on the laser focal depth.

However, in the above cited works the NPs produced using top-hat [137] and Bessel beams [138] had a spherical shape. On the contrary, a very recent work by Marrapu et al. [4] demonstrates silver nanoribbons produced using cylindrical ps laser beam ablation of silver in water. Interestingly, in the conditions used, the nanoribbons were observed only for one specific laser energy of ≈1200 μJ, while both higher and lower pulse energies produced only spherical NPs. The authors attributed this observation to the co-melting of preliminarily formed silver NPs in the cylindrical laser beam which directed the welding into one-dimensional ribbons. Alternatively, the conditions in the cavitation bubble, its dynamics, size, and pressure as well as its oscillations are considered responsible for the nonspherical morphology in the cited work. Similar assumptions are made in [138]. When the laser beam pattern deviates from a Gaussian profile, it induces pressure variations at the target interface, which become a key factor in determining the morphology of the resulting nanomaterials. Moreover, the shockwave dynamics would also be influenced, which would alter the processes occurring in the cavitation bubble and determine the morphology and structure of the forming nanomaterials.

The other shapes of interest towards nonspherical NP formation are annular and vortex. The annular beam shape can be formed by illuminating the axicon and convex lens using a Gauss beam. The vortex-shaped beams have been already intensively applied for structuring of surfaces and formation of nonspherical nanostructures, including nanoneedles, nanodomes, spiralling nanofibers, nanodisks, and 3D chiral nanostructures, which have been demonstrated for various materials: Si [152–153], Ta [154–155], Ag [156], Al [157], graphene [158], and others. In a liquid, all the ablation processes would be influenced by the additional confinement introduced by a liquid. The shape of the formed structures can be varied upon change of liquid parameters, namely temperature and viscosity [139]. Moreover, in the case of laser ablation in liquid, the formation of microjets is reported [159–160]. The works in this direction are urgently required to understand the possibilities to control NP morphology and structure.

### 4 Challenges and prospects of shape-controlled synthesis of nanostructures

The tailoring of NP shapes represents an important task in the laser ablation field, although it is still a challenge. The major approaches developed for shape control are summarized in the Table 1.

**Table 1:** Summary of the major approaches and experimental parameters used for the synthesis of nonspherical nanostructures by laser-assisted methods.

NPs	Shape	Liquid	Target	Method	Laser wavelength, nm	Pulse duration	Comment	Ref

Pulsed laser ablation in liquids								

ZnO	nanorods	NH_4_NO_3_ solution	Zn	PLAL^a^	1064	ns	shape influenced by LIM and subsequent Ag ablation	[2]
Ag_2_O	mixture of cubes, pyramids, triangular plates, penta-gonal rods, and bars	polysorbate 80 aqueous solution	Ag	PLAL	248	ns	excimer laser	[3]
Ag	nanoribbons	double distilled water	Ag	PLAL	800	ps	cylindrical focusing	[4]
Ag	polygonal particles	acetone	Ag	PLAL	1045 and 1064	fs, ns	spontaneous shape transformation during ageing	[14]
Mn_3_O_4_	nanocubes	water, water–ethanol mixture	Mn	PLAL	1064	ps	–	[18]
FeMn@ FeMn_2_O_4_	core–shell NPs	ethanol	FeMn	PLAL	1064	ps	–	[18]
MnO*_x_*	nanosheets, nanofibers	water, water–ethanol mixture	FeMn	PLAL	1064	ps	–	[18]
Te	nanochains	water	Te	PLAL	1064	ns	transformation to spherical aggregates after ageing	[19]
Te	nanochains	methanol	Te	PLAL	1064	ns	transformation to microspheres after ageing	[19]
Te	nanochains	ethanol	Te	PLAL	1064	ns	transformation to microspheres after ageing	[19]
Te	nanochains	acetone	Te	PLAL	1064	ns	transformation to microspheres after ageing	[19]
Te	nanocubes	CH_2_Cl_2_	Te	PLAL	1064	ns	transformation from nanochains to nanocubes upon ageing	[19]
TiO_2_	1D super-structure	tetraethyl orthosilicate	Ti	PLAL	–	–	nanocrystal growth via oriented attachment	[20]
YVO_4_ :Eu^3+^	ovoid-like particles	deionized water	Y_0.95_VO_4_: 0.05Eu^3+^	PLAL	532	ns	oriented attachment assisted by acoustic wave generated by PLAL	[21]
GaOOH	spindle-like	CTAB aqueous solution	Ga	PLAL	1064	ns	–	[22]
GaOOH	irregularly shaped particles	SDS aqueous solution	Ga	PLAL	1064	ns	–	[22]
GaOOH	irregularly shaped particles	water	Ga	PLAL	1064	ns	–	[22]
PbO	nanosheets	water	Pb	PLAL	1064	ns	nanosheets formed after ageing	[26]
ZnS	nanowires	dodecyl mercaptan	Cu/Zn	PLAL	1064	ms	Cu nanodroplets catalysis growth of nanowires	[29]
PbS/ZnS	PbS tipped ZnS nanorods	dodecyl mercaptan	Zn/Pb	PLAL	1064	ms	liquid influence, formation of heterostructures if pulse duration >10 ms	[25]
Mn-doped Ni(OH)_2_	nanosheets	NiCl_2_ aqueous solution	Mn	PLAL	1064	ns	Self-assembly of nanosheets into hierarchical structures	[30]
ZnO	nanosheets	deionized water	Zn	PLAL	1064	ns	dependence of shape on laser pulse energy and wavelength, spherical NPs at 532 nm ablation	[32]
ZnO	drop-shaped	methanol	Zn	PLAL	1064	ns	self-assembly into dendritic nanostructures	[33]
Bi-based NPs	flake-like nanosheets	water	Bi	PLAL	1030	fs	–	[34]
CuO	nanospindles	water	Cu	PLAL	1064	ns	shape transformation from spherical to nanospindles upon ageing and temperatures ≥35 °C	[36]
Zn(OH)_2_/SDS	layered zinc hydroxide/ dodecyl sulfate hybrid nanosheets	SDS solution	Zn	PLAL	1064	ns	layered structures explained by thermal vaporization of Zn species in SDS solutions	[38]
Ag	nanowires	sodium citrate, PVP solutions	Ag	PLAL	800	fs	effect of polarization	[39]
AgBr-based	nanosheets	cetyltrimethyl- ammonium bromide (CTAB) solution	Ag	PLAL	355	ns	assembly with surfactant molecules by charge-matching mechanism to produce inorganic–organic nanocomposite	[40]
ZnO	nanoflakes	distilled water	Zn	PLAL	532	ns	dependence of the shape on the solution temperature and laser fluence	[46]
Au	nanowires, nanofilaments	superfluid He	Au	PLAL	532	ns	PLAL in superfluid gases	[48]
metals (Au, Cu, Cs, Ba, Rb)	nanofilaments	liquid He	metals (Au, Cu, Cs, Ba, Rb)	PLAL	532 or 355	ns	PLAL in superfluid gases	[49]
refracttory metals (Nb, Re, W, Mo)	nanowires	superfluid He	metals	PLAL	1.06 µm	ns	PLAL in superfluid gases	[50]
CuO	nanosheets	H_2_O_2_ solution	Cu	PLAL	1064	ns	morphology depend on H_2_O_2_ concentration and laser pulse energy	[54]
Al_2_O_3_	hollow micro/ nanoparticles	water	Al	PLAL	248	ns	excimer laser ablation, addition of ethanol favours hollow particle formation	[56]
ZnO_x_	hollow NPs	water/ethanol mixture	Zn	PLAL	–	–	excimer laser, self-assembly at the cavitation bubble interface	[57]
Al, Ti	porous NPs	hydrogen- saturated ethanol	Al, Ti	PLAL	800/1064	fs/ns	saturation of liquid with gaseous hydrogen, dependence on the laser pulse duration	[58]
Au	nanowires	double distilled water	Au	PLAL	355, 532, 1064	ns	influence of laser wavelength on NP shape	[75]
Au	nanowire networks	water	Au	PLAL	355, 532	ns	shape determined by laser fluence and wavelength	[76]
β-MnO_2_	nanowires	deionized water	Mn	PLAL	532	ns	spontaneous assembly, high aspect ratio nanowires after annealing at 300 °C for 3 h	[90]
WO_3_	porous nanoflakes	deionized water	W	PLAL	532	ns	porous nanoflakes formed after annealing at 800 °C for 4 h	[91]
Gd_2_O_3_	nanorods, nanoflakes	distilled water	Gd_2_O_3_	PLAL	1064, 532	ns	dependence on wavelength and fluence: nanorods are dominant at 1064 nm ablation, nanoflakes – at 532 nm	[123]
Ag	near-spherical	deionized water	Ag	PLAL	532	ns	shape variation with increasing laser fluence	[125]

Laser ablation/irradiation of microstructures								

CuO/Cu	irregularly shaped particles	ethyl acetate	CuO powder	PLAL	532	ns	–	[16]
ZnSe	nanowires and partially hollow nanotubes	deionized water	ZnSe micro- particles	PLAL	800	fs	self-assembly of NPs results in nanowires, introduction of air produce nanotubes	[31]
FeOOH	nanowires	methanol	Fe powder	PLAL	248	ns	transformation of NPs to nanowires under 248 nm irradiation	[28]
Cu	nanowires	methanol, ethanol	Cu micro- flakes	PLAL	800	fs	effect of laser polarization	[126]
[Ni-Fe]-layered double hydroxides	nanosheets	nitrate solutions	Fe, Ni powders	laser irradiation	355	ns	impact of the ablation target, metal ion type and concentrations, and laser pulse energies	[51]
MoO_3−_*_x_*	nanosheets	ethanol/ water	MoS_2_ powders	laser irradiation	800	fs	nanosheet formation depended on ethanol concentration	[52]
multilayered graphene, WS_2_, MoS_2_, and BN	nanorods	water-ethanol	graphene, MoS_2_, WS_2_, and BN flakes	LIM	800	fs	linear polarization promotes the formation of nanorods	[132]
MoS_2_	nanosheets	FeCl_3_ solution	MoS_2_ crystal	laser exfoliation	800	fs	laser-induced exfoliation with doping by Fe^3+^	[53]

Laser-induced modification in liquids								

Ag	nanowires	Ag aqueous colloid	–	LIM^b^	355	–	fusion of NPs	[74]
Ag	nanowires	Ag colloid	–	LIM	532, 266, 400 and 800	ns	dependence on the laser wavelength	[65]
Ag	microbelts	Ag NPs in ethanol	–	LIM	532	ns	aggregation of NPs with fusion results in 1D structures	[77]
Ag	nanocubes	Ag NPs in acetone–water solution	–	LIM	355	ns	spontaneous atom transportation process	[64]
Ag	nanodisks/ triangles	Ag NPs colloid with additional AgNO_3_ and citrate	–	LIM	457/ 514	–	linearly polarized laser beam, photoreduction catalysed by Ag NPs	[78]
Au	nanothreads	Au NPs colloid	–	LIM	805	fs	scaffolding with cucurbit[7]uril (CB) molecules	[84]
Cu	nanowires	Cu nanoflakes in ethanol	–	LIM	780	fs	linear polarization	[85]
Cu	nanowires	Cu flakes in methanol	–	LIM	780	fs	linear polarization	[86]
Ag	nanowires	Ag nanospheres in ethanol/PVP	–	LIM	532	ns	polyvinyl- pyrrolidone capping agent favours the assembly and shape transformation	[87]
Au	nanowires	Au NPs colloid with PVP	–	LIM	532	ps	impact of the excessive charge on NP surfaces	[88]
ZnO	nanorods	ZnCl_2_-NH_4_OH	–	LIM	780	fs	heterogeneous nucleation with subsequent hydrothermal process	[126]

Laser irradiation of interfaces								

Au–Ag supramolecular complex	nanoparticles, 2D flakes, multipetal “flower-like” structures	supramolecular complex^c^ in acetone, aceto-phenone, or dichlor-ethane	–	laser- induced deposition from solution	325	CW	laser irradiation of the substrate–solution interface	[80]
hybrid carbon–metal structures	nanoflakes	supramolecular complex^c^ in acetone, aceto-phenone, or dichlor-ethane	–	laser- induced deposition from solution	325	CW	laser irradiation time and applied electric field influence the shape of flakes	[81]
Ag	nanofibers	silver benzoate water solution	–	laser- induced deposition from solution	448	CW	laser irradiation time increases the width and length of nanofibers	[82]
Ag	nanofibers	silver benzoate hydrate solution	–	laser- induced deposition from solution	266, 374, 405, 448	CW	self-template synthesis	[83]

External field-assisted laser-ablation in liquids								

ZnO	nanoflowers	distilled water	Zn	EFLAL^d^	1064	ns	electric field applied to target, shape depends on the applied voltage	[11]
ZnO/C	porous sponge-like structure	C NPs aqueous colloid	Zn	EFLAL	1064	ns	electric field applied to target	[11]
Au	elongated	NaOD in D_2_O	Au	EFLAL	532	ps	NP synthesis in β-active liquids with and without cathodic bias	[88]
CuO	nanospindles	deionized water	Cu	EFLAL	532	ns	shape determined by applied voltages	[96]
Au	nanospheres, nanocubes, nanospindles, rhombi, triangles	deionized water	Au	EFLAL	1064	ns	shape and size depended on the electric field value	[101]
Pt	spherical, hexagon, and rectangular shapes	distilled water	Pt	EFLAL	1064	ns	nonspherical particles observed at specific electric field strengths	[103]
Bi_2_O_3_	irregular shapes	distilled water	Bi	EFLAL	1064	ns	influence of the electric field strength on the shape and size of nanoparticles	[108]
GeO_2_	micro- and nanocubes, nanospindles	deionized water	Ge	EFLAL	532	ns	electric field influence on the shape of NPs	[109]
Ag	cube, cuboid, hexagon, and pentagon	distilled water	Ag	EFLAL	1064	ns	the NPs of polygonal shapes in electric fields of 100 V·cm^−1^ and 1000 V·cm^−1^	[110]
ZnO/C	hierarchical nanostructures	C NPs colloid	Zn	EFLAL	1064	ns	assembly on cathode into hierarchical structures, dependence of the shape on the applied field direction and strength	[111]
TiO_2_	elongated	NaOH solution	Ti	EFLAL	532	ps	cathode bias	[112]
Ge	spherical, spindle, nanowebs	distilled water or ethanol	Ge	EFLAL	351	ns	shape dependence on liquid, applied voltage	[120]
ZnMoO_4_	nanoflowers	deionized water	Mo	ECLAL	532	ns	formation of nanostructures from material of Mo target and Zn electrodes	[97]
copper vanadates	nanoflakes, nanoflowers, nanoplates	deionized water	V	ECLAL^e^	532	ns	Cu electrodes, the shape depends on the applied voltage	[98]
CuMoO_4_	nanospindles, cuboids	deionized water	MoS_2_	ECLAL	800	ps	Cu electrodes inject Cu ions into the plasma	[99]
Au	nanorods	water	Au	MFLAL^f^	1060–1070	ns	elongation under an external magnetic field	[92]
Co_3_C	nanochains	ethanol	Co	MFLAL	532	ns	magnetic-field- assisted laser ablation in liquid combined NP production with chain fabrication	[93]
FePt	nanochains	ethanol	Fe-based alloys	MFLAL	532	ns	–	[94]

Combined methods								

Bi_2_O_3_	nanosheets	water	Bi	ultrasound- assisted LAL	355	ns	ultrasound promotes self-assembly into nanosheets	[61]
AgGe	football-like microspheres	Ge NPs in AuCl_3_–AgNO_3_ aqueous solution	–	PLAL+ LIM	1064, 532	ps	Ge NPs favour seed-mediated growth	[23]
Zn_2_GeO_4_	nanowires	deionized water	Zn, Ge	PLAL+ hydrothermal	1064	ns	dual laser–hydrothermal method: simultaneous ablation of Zn and Ge targets followed by hydrothermal growth	[89]

^a^PLAL – pulsed laser ablation in liquid; ^b^LIM – laser-induced modification, ^c^Supramolecular complex – [Au_13_Ag_12_(C_2_Ph)_20_(PPh_2_(C_6_H_4_)_3_PPh_2_)_3_][PF_6_]_5_, ^d^EFLAL – electric-field-assisted laser ablation in liquid, ^e^ECLAL – electrochemistry-assisted laser ablation in liquid, ^f^MFLAL – magnetic-field-assisted laser ablation in liquid.

The control of NP properties, such as size and shape, is possible both at the stages of NP formation and growth. However, it requires further insights into the fundamental processes and mechanisms occurring at each stage of the complex process of laser ablation in a liquid. The impact of plasma parameters, such as composition, temperature, and pressure on the produced NP composition, size, and shape remains nearly unstudied. Similarly, the literature lacks studies on the dependence of NP shape on the cavitation bubble pressure and shockwave generation and propagation. However, these parameters can be crucial and determine the process of NP formation.

Furthermore, the simultaneous influence of multiple parameters on nanoparticle shape makes it much more difficult to understand how experimental conditions can be varied to control morphology. The scientific aspects of the interplay between different experimental parameters influencing shape need to be studied, which requires both simulations and experimental assessment. This is especially urgent for novel methods of laser ablation under external electric fields, where the information on the plasma and cavitation bubble evolution is missing. This hinders its development into a precise shape-controlled synthesis method and limits its application, despite all the intriguing and promising results already demonstrated. As a result, in many cases, mixtures of different shapes are produced. In addition, upon action of high laser powers or longer ablation times, further conversion of the materials shape can occur, which is still not well understood in the literature. The multitude of chemical processes occurring in plasma, cavitation bubble, and liquid as well as interfaces, and with the variety of active short-living species necessitates further studies of the induced chemistry as well. This is especially relevant for the formation of complex compound and composite nanostructures.

Practically not studied is the impact of laser focusing conditions on the nanoparticle shapes. The application of non-Gaussian beam shapes, such as Bessel or annular, can provide multiple benefits for laser ablation synthesis, including formation of different shapes. However, further development in this directions requires careful studies of the occurring processes and comparison of the results with the synthesis in conventional conditions.

This aspect implies further upgrade of the developed models and experimental methods of laser ablation processes. The challenge associated with the experimental evidence and confirmation of the suggested mechanisms has always existed in laser ablation research due to fast evolution and transient nature of the occurring processes involved in a laser ablation event. The imaging of cavitation bubble dynamics is usually possible only using the intensified charge coupled device sensors, providing imaging possibilities with a nanosecond temporal resolution. For shockwave observation, the methods of shadowgraphy or Mach–Zehnder interferometry are usually applied. However, the imaging of shockwaves is challenging at early stages when the emission of the plasma plume is intense. Therefore, in most cases, the shockwave imaging is studied only after several hundreds of ns after plume generation.

The addressing of these challenges will determine the future trends in the field of shape-controlled synthesis of NPs by PLAL. It can be expected that the key efforts in the field will be related to the understanding of NP formation. The insights into the PLAL mechanisms will aid in further optimizing the process parameters (laser pulse duration, wavelength, fluence, focusing conditions, and liquid composition) to control NP morphology, composition, and increased ablation rate. As a result, the research will be directed towards achieving specific NP structure and characteristics, expanding the applicability of PLAL to more complex anisotropic structures and designs targeted for advanced applications.

Fundamental insights and advanced diagnostics are urgently required for further development of external-field-assisted PLAL in liquids, which can be considered as one of the future trends in the synthesis of nanostructures. Currently, the electric-field-assisted synthesis is gaining an increased attention both for synthesis and assembly of NPs. However, the application of magnetic and temperature fields and gradients can be of interest as well resulting in tailored synthesis approaches.

The application of laser ablation using different beam shapes will also be intensified considering the recent results demonstrating the benefits for the control of both morphology and size of nanostructures. A very recent work, for example, demonstrates the application of donut-shaped laser beam for laser ablation in liquid, and despite that only nanoparticles of spherical shape were produced, the authors admit the substantial role of the beam shape on narrowing the size distribution [130].

Another direction is the development of combined and multistep laser-assisted approaches. The method of sequential laser ablation and laser ablation combined with laser-induced fragmentation or melting of the nanoparticles in colloids has already been demonstrated as a convenient method of complex NP formation and tailoring, as shown above. Apart from purely laser-assisted approaches, combining PLAL with other techniques, such as hydrothermal synthesis, ultrasonic, or plasma treatment, is opening up new avenues for nanomaterial synthesis. The advanced combined methods can also expand the material diversity towards multicomponent, alloyed, doped, and composite nanostructures targeted for specific application. Furthermore, the development of combined synthesis and deposition methods can be expected, providing the pathways of assembly of as-synthesized nonspherical nanostructures into complex hierarchical structures, which are important for certain applications including energy and catalysis. Overall, the PLAL method holds significant potential for the future of nanomaterial synthesis, with ongoing research addressing key challenges and expanding the scope for its applications.

## Conclusion and Outlook

To conclude, the laser ablation method has reached significant progress in understanding the occurring processes and mechanisms, and it is about to make the next step towards versatile sustainable nanofabrication methods by applying targeted nanomaterials at a large scale. These novel fundamental insights allowed better control over production efficiency, size, and composition of prepared NPs to expand the application areas and variety of nanomaterials synthesized using the PLAL method. However, control and tailoring of nanoparticle shapes have always been challenging tasks. Nevertheless, some insight into the processes and key parameters influencing NP shape has been achieved, allowing for the development of several strategies for the formation of one- and two-dimensional nanostructures, nanocubes, nanospindles, among others. Furthermore, conditions towards novel dendritic and hierarchical nanostructures were found, which are on high demand for practical applications. In all cases, manipulation of surface charges is shown to be a perspective strategy for shape tuning. This can be achieved by a multitude of methods, including application of external electric fields, utilization of polarized laser light, or charged species in the solution. As a result, the application of external fields appears to be the most promising way for the controlled production of nanostructures with a desired shape. The applied electric fields provide ways to control size, shape, and morphological parameters, such as aspect ratio of elongated nanostructures. This is done by changing the magnitude and direction of the electric field in addition to the laser parameters and liquid properties, resulting in a promising nanoscale manufacturing technique. The initiation of electrochemical processes in solution combined with the effect of nonspherical nanostructure assembly on the electrode is opening a pathway for one-pot synthesis of hierarchical nanostructures with a complex composition for applications in energy storage and catalysis. The variation of liquid composition can be used for surface charge engineering of the growing nanostructures resulting in morphological and structural changes. For this purpose, the addition of surfactants or ions is done, which enables oriented growth guided by the attached surfactant molecules or adsorbed ions, or self-assembly into solution.

The works demonstrating the approaches to control nanostructure shape or to initiate laser-induced shape change are increasing, which ensures the progress in the field of pulsed laser synthesis of nanomaterials in liquid going beyond common near-spherical NPs.

## Data Availability

Data sharing is not applicable as no new data was generated or analyzed in this study.
